# Genetic interaction involving photoperiod-responsive *Hd*1 promotes early flowering under long-day conditions in rice

**DOI:** 10.1038/s41598-018-20324-1

**Published:** 2018-02-01

**Authors:** Prasanta K. Subudhi, Teresa B. De Leon, Ronald Tapia, Chenglin Chai, Ratna Karan, John Ontoy, Pradeep K. Singh

**Affiliations:** 1 School of Plant, Environmental, and Soil Sciences, Louisiana State University Agricultural Center, Baton Rouge, LA 70803 USA; 2 University of California, Davis, CA USA; 3 Department of Horticultural Science, University of Florida, IFAS Gulf Coast Research and Education Center, 14625 CR 672, Wimauma, FL 33598 USA; 4 Noble Research Institute, 2510 Sam Noble Parkway, Ardmore, OK 73401 USA; 5 University of Florida, Gainesville, FL 32611 USA; 6 Division of Genetics, Indian Agricultural Research Institute, New Delhi, 110012 India

## Abstract

Although flowering in rice has been extensively investigated, few studies focused on genetic interactions. Flowering evaluation of two recombinant inbred line (RIL) populations involving photo-insensitive rice cultivars, Bengal and Cypress, and a weedy rice accession, PSRR-1, under natural long-day (LD) conditions, revealed six to ten quantitative trait loci (QTLs) and a major QTL interaction. In addition to the validation of several previously cloned genes using an introgression lines (IL) population of PSRR-1, a few novel QTLs were also discovered. Analysis of the marker profiles of the advanced backcross lines revealed that *Hd1* allele of PSRR-1 was responsible for the photoperiodic response in the near-isogenic lines (NILs) developed in both cultivar backgrounds. Based on the phenotypic and genotypic data of the NILs, and NIL mapping population and the transcript abundance of key flowering pathway genes, we conclude that *Hd1* and its interaction with a novel gene other than *Ghd7* play an important role in controlling flowering under LD conditions. Our study demonstrates the important role of genetic interaction that regulates flowering time in rice and the need for further investigation to exploit it for breeding adaptable rice varieties.

## Introduction

Flowering time is a complex agronomic trait governed by both genetic factors and environmental cues^1,2^. Variation in day length is an important environmental signal that regulates flowering in many plants^3^. Based on the flowering response to day length variation, plants are classified as long-day (LD), short-day (SD) or day neutral plants. As an important cereal crop, rice is grown in places with wide variation in photoperiod all over the world^4,5^. Despite the progress made in deciphering the molecular mechanisms involved in the flowering response, there are many fundamental unanswered questions due to the genetic complexity of this trait^6^. Particularly, the molecular basis of the wide range of genetic variation, the coordination of the different downstream genes in regulatory networks, and the genes regulating these regulators are still not clear.

In rice, two independent flowering pathways have been recognized: *HEADING DATE 1* (*Hd1*)-dependent pathway and *EARLY HEADING DATE 1* (*Ehd1*)-dependent pathway^7–10^. *Hd1*, an ortholog of *CONSTANS (CO)* of *Arabidopsis*, promotes flowering under SD conditions but strongly represses flowering under LD conditions through regulation of the expression of *Hd3a*^7,11^. On the other hand, *Ehd1* promotes flowering under both SD and LD condition, but its effect is stronger in promoting flowering under SD condition by activating *Hd3a* and its paralog *RFT1*^9,10,12^. Both *Hd3a* and *RFT1* encode the mobile flowering signal proteins, which are essential for flowering initiation^13^. *Ehd1* is both positively and negatively regulated by a number of genes^14–22^. Among these, *Ghd7* is an important member of the flowering pathway that regulates plant height, heading date, and grain number^16^. It delays flowering under LD by repressing *Ehd1* transcription.

Diversity in flowering time in rice varieties is largely due to presence of diverged alleles of the flowering genes and their interactions^12,23–28^. *LH8* encoding a CCAAT-box-binding transcription factor with *Hd1*-binding activity delayed flowering by repressing the expression of *Ehd1*^29^. Similarly, the physical interaction between *Heading date Associated Factor 1 (HAF1)*, a C3HC4 RING domain-containing E3 ubiquitin ligase, and *Hd1* influenced photoperiodic flowering response through regulation of *Hd1* accumulation^30^. *Ghd7*, a key floral repressor gene with major influence on rice yield^16^, was reported to influence the function of *Hd1* in delaying or promoting flowering under long-day condition^31^. The binding of the protein complex formed by the CCT domain of Ghd7 protein and the transcription activating domain of Hd1 protein to the regulatory region of *Ehd1* led to its repression and florigen genes under LD condition^32^. Another study indicated that the adaptation to specific agroclimatic region and yield potential depended on the combinations of *Ghd7, Ghd8*, and *Hd1* in rice varieties^33^. Since the time of transition from the vegetative to flowering stage is vitally important for maximizing productivity, elucidation of the new genetic determinants and their interactions controlling this transition is essential to breed new high yielding rice varieties adapted to a specific cropping season or agroclimatic region.

The current study focused on the elucidation of the genetic interaction involved in the flowering transition in response to photoperiod using unique genetic materials such as recombinant inbred line (RIL) and introgression line (IL) populations, and near-isogenic lines (NILs) developed from crosses involving two photo-insensitive cultivars and a weedy rice accession. We discovered that the *Hd1* from the weedy rice accession in a cultivated rice background exhibited late flowering under LD condition. We further demonstrated that early flowering and photo-insensitivity in weedy rice was due to genetic interaction between *Hd1* and a novel gene other than *Ghd7* on chromosome 7.

## Results

### QTL mapping for heading date in BR and CR RIL populations

All three parents were photo-insensitive, but the hybrids of the BR (Bengal x PSRR-1) and CR (Cypress x PSRR-1) crosses were highly photosensitive in the natural field environment. Although the difference in mean days to heading (DTH) between both populations was around 10 days, the range was wide in each population with some transgressive segregants flowering earlier and later than either parent (Supplementary Fig. S1). In both populations, the distribution was skewed toward earlier flowering. A majority of RILs flowered within 70–100 and 80–130 days in the BR and CR populations, respectively^34^.

**Figure 1:**
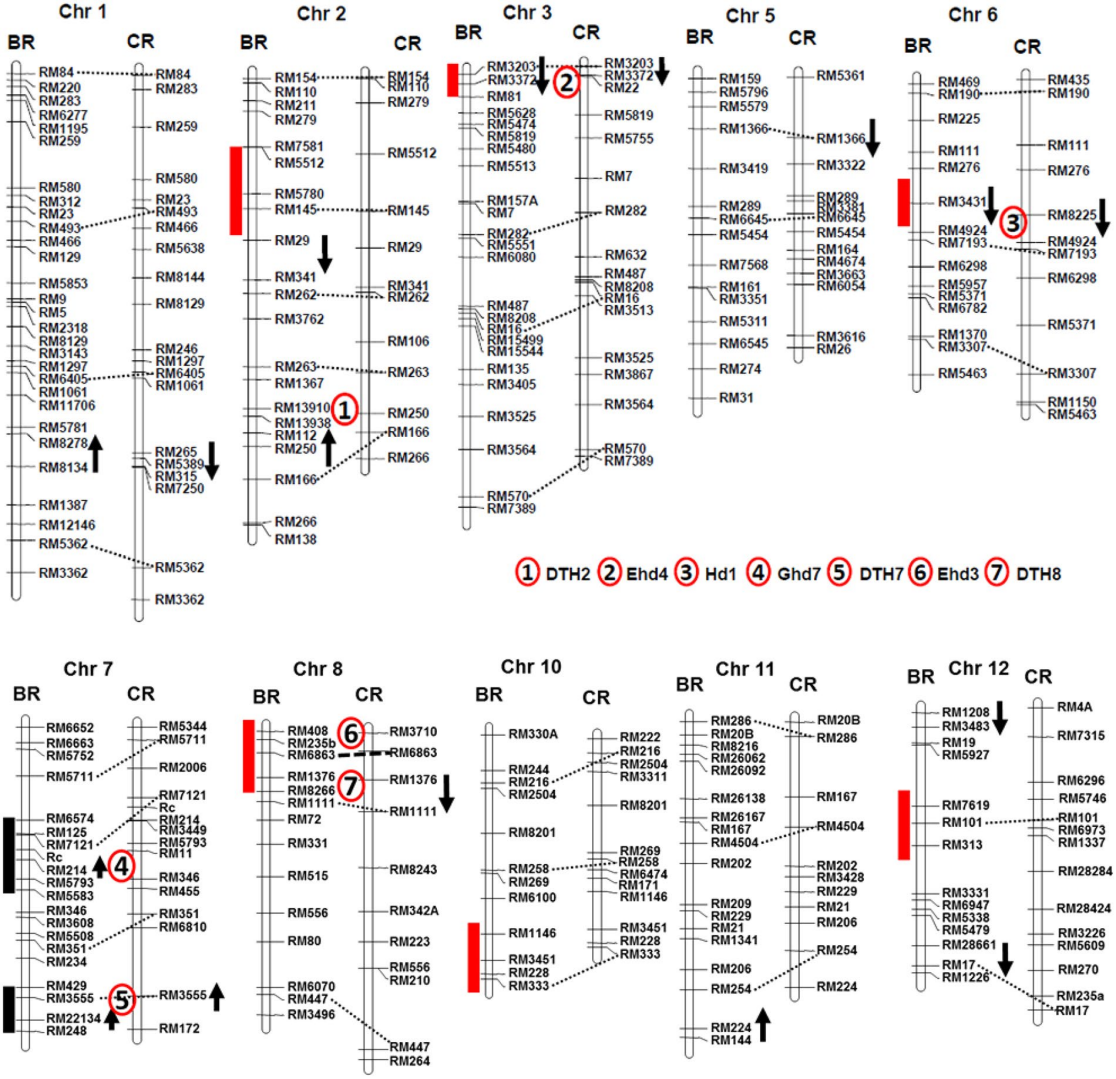
Map location of the QTLs for heading date in the RIL populations developed from the crosses, Bengal x PSRR-1 (BR) and Cypress x PSRR-1 (CR)^34^ and IL population of ‘PSRR-1’ developed in ‘Bengal’ background^35^. Linkage groups in both RIL populations were aligned using common markers. The arrows were placed in 1-LOD interval and arrows pointing to the top and bottom indicate increasing and decreasing effect on phenotypic values of the ‘Bengal’ or ‘Cypress’ allele, respectively. The solid bars to the left of chromosomes of BR linkage map indicate the introgressed PSRR-1 chromosome segments in ILs. The solid bars were red or black when the PSRR-1 alleles increased or decreased trait means in ILs compared to the recurrent parent, respectively. The presence of QTL was inferred when there was a significant difference between the mean of an IL and the recurrent parent at P < 0.01 using the Dunnett’s test. Heading date genes overlapping the QTLs identified in this study were indicated by numbers 1-7 (1 = *DTH2*, 2 = *Ehd4*, 3 = *Hd1*, 4 = *Ghd7*, 5 = DTH7, 6 = *Ehd3*, 7 = *DTH8*).

Ten QTLs were detected on 7 chromosomes accounting for 58% of phenotypic variation in the BR-RIL population (Table 1, Figs 1, and S2). There were 2 QTLs each on chromosomes 2, 7, and 12 whereas chromosomes 1, 3, 6, and 11 harbored only one QTL. A wide range of variation was observed with respect to the magnitude of additive effects and percentage of the phenotypic variation explained by each QTL. The ‘Bengal’ and ‘PSRR-1’ alleles were responsible for increased DTH in case of five QTLs each. For the largest effect QTL *qHD7–1*^*BR*^ (R^2^ = 31%), the ‘Bengal’ allele increased the DTH, whereas the contribution of each of the remaining QTL accounted for 3–9% of the phenotypic variation. After removing the highly photosensitive lines, the same QTLs on chromosomes 1, 3, 6, and 7 were detected with similar additive effects but not those on chromosomes 2, 11, and 12 (data not shown).

**Table 1:** Quantitative trait loci for heading date in BR-RIL population detected using a composite interval mapping procedure.

QTLs	Marker interval	Physical size of QTL (Mb)	Position^a^	LOD	AE^b^	PVE^c^	Increasing effect^d^
*qHD1* ^*BR*^	RM8278-RM8134	2.278	128.9	3.6	3.94	4.1	Bengal
*qHD2-1* ^*BR*^	RM29-RM341	9.032	58.3	6.0	−4.93	6.4	PSRR-1
*qHD2-2* ^*BR*^	RM112-RM250	0.761	126.2	3.6	3.26	3.0	Bengal
*qHD3* ^*BR*^	RM3203-RM3372	0.659	3.0	3.6	−4.87	3.1	PSRR-1
*qHD6* ^*BR*^	RM3431-RM4924	9.728	45.9	9.2	−5.79	8.5	PSRR-1
*qHD7-1* ^*BR*^	Rc-RM214	6.721	46.0	28.0	13.16	31.0	Bengal
*qHD7-2* ^*BR*^	RM22134-RM248	0.591	102.4	6.3	4.93	5.9	Bengal
*qHD11* ^*BR*^	RM224-RM144	0.608	109.3	3.2	3.17	2.6	Bengal
*qHD12-1* ^*BR*^	RM1208-RM3483	1.612	1.0	3.1	−3.14	2.7	PSRR-1
*qHD12-2* ^*BR*^	RM28661-RM17	1.558	88.5	3.8	−3.73	3.8	PSRR-1
					Total^e^	58.3	

^a^QTL peak position on the linkage map.

^b^Additive effects of ‘Bengal’ allele.

^c^Phenotypic variation (%) explained by each QTL.

^d^Source of allele increasing the trait mean.

^e^Estimate of the total phenotypic variation explained by the QTL from a multiple QTL model fit in QTL Cartographer^43^.

In the CR-RIL population, six QTLs on chromosomes 1, 3, 5, 6, 7, and 8 explained 47% of the phenotypic variation (Table 2, Figs 1, and S3). The contribution of each QTL ranged from 3 to 29%. The ‘Cypress’ allele increased DTH in case of *qHD7*^*CR*^ and ‘PSRR-1’ allele increased in the rest. The *qHD3*^*CR*^ with the largest effect explained 29% of phenotypic variation, whereas *qHD7*^*CR*^ explained 14% of the phenotypic variation. After removing the highly photosensitive lines, the same QTLs were detected with similar magnitude of additive effects (data not shown).

**Table 2:** Quantitative trait loci for heading date in the CR-RIL population detected using a composite interval mapping procedure.

QTLs	Marker Interval	Physical size of QTL (Mb)	Position^a^	LOD	AE^b^	PVE^c^	Increasing effect^d^
*qHD1* ^*CR*^	RM265-RM5389	0.536	136.3	2.5	−4.11	2.5	PSRR-1
*qHD3* ^*CR*^	RM3203-RM3372	0.659	3.0	20.5	−14.57	29.1	PSRR-1
*qHD5* ^*CR*^	RM1366-RM3322	1.346	25.2	3.6	−4.76	3.7	PSRR-1
*qHD6* ^*CR*^	RM276-RM8225	3.079	56.3	3.1	−5.05	3.7	PSRR-1
*qHD7* ^*CR*^	RM3555-RM172	1.671	114.5	10.1	9.09	13.7	Cypress
*qHD8* ^*CR*^	RM1376-RM1111	1.605	24.8	5.1	−6.21	6.8	PSRR-1
					Total^e^	47.2	

^a^Peak position of the QTL on the linkage map.

^b^Additive effects of ‘Cypress’ allele.

^c^Phenotypic variation (%) explained by each QTL.

^d^Source of allele increasing the trait value.

^e^Estimate of the total phenotypic variation explained by the QTL from a multiple QTL model fit in QTL Cartographer^43^.

The comparison of the QTL positions in both populations revealed four consistent QTLs on chromosomes 1, 3, 6, and 7 despite the variation in the direction of allelic effect on heading date variation (Fig. 1). The QTL on chromosome 1 was congruent, but the allelic effect was in opposite direction in both populations. The ‘PSRR’ allele enhanced DTH at QTLs on chromosomes 3 and 6, whereas the cultivated allele was responsible for increasing DTH at QTL on chromosome 7.

### QTL mapping in IL population of ‘PSRR-1’ in ‘Bengal’ background

Evaluation of a genome-wide IL population of PSRR-1^35^ indicated that 92% of ILs had DTH similar to the recurrent parent (RP) (Supplementary Fig. S4). Three ILs with introgressed segments of chromosome 7 flowered earlier than ‘Bengal’ and nine ILs with ‘PSRR-1’ segments from chromosomes 2, 3, 6, 8, 10, and 12 exhibited late heading compared with RP (Supplementary Table S1). When overlapping of introgressed segments was analyzed, six genomic regions responsible for increasing DTH and two genomic regions for decreasing DTH were identified (Fig. 1). The coincident QTLs located on chromosomes 3, 6, and 7 in both populations could be validated in these ILs. In addition, a few QTLs present in either BR (*qHD2-1*, *qHD7-1)* or *CR* populations (*qHD8)* were also confirmed. Among all these ILs with significantly different DTH compared to RP, the chromosome 6 IL was extremely late and photosensitive with the largest effect. However, it was detected as a minor QTL in both RIL populations. For both QTLs on chromosome 7, ‘Bengal’ alleles increased DTH, which was consistent with the observation that the ILs harboring this region showed early heading compared to ‘Bengal’.

Based on the physical location of the cloned flowering genes and the molecular markers, we could locate several of them on the linkage map. Those overlapping with the QTLs were *Ehd4* (*Hd2*) on chromosome 3, *Hd1* on chromosome 6, *Ghd7* (*Hd4*) and *DTH7* on chromosome 7, and *Ehd3, DTH8 (Hd5 or Ghd8*) on chromosome 8 (Fig. 1).

### Effect of photoperiod on DTH on parents and photosensitive NILs

Both BRNIL-20 and CRNIL-58 showed strong photosensitivity (PS) with initiation of flowering in 160–165 days. Molecular marker profiles of these lines indicated that both NILs had a ‘PSRR-1’ segment on chromosome 6 (RM225-RM4924 in BRNIL-20 and RM8225-RM5371 in CRNIL-58) (Supplementary Fig. S5). Based on the physical map locations of the flanking markers in both NILs and of *Hd1*, we concluded that *Hd1* was located on the introgressed PSRR-1 segments of both NILs but not *Hd3a* and *RFT1*. Using the principle of substitution mapping^36^, the location of *Hd1* was narrowed down to the RM3431-RM4924 and RM8225-RM4924 regions in BRNIL-20 and CRNIL-58, respectively (Supplementary Fig. S5). The QTL results showing the peak position of this QTL in both BR and CR-RIL populations at RM3431 (8.74 Mb) and RM8225 (9.31 Mb), respectively (Fig. 1), provided further evidence regarding *Hd1* involvement.

**Figure 2:**
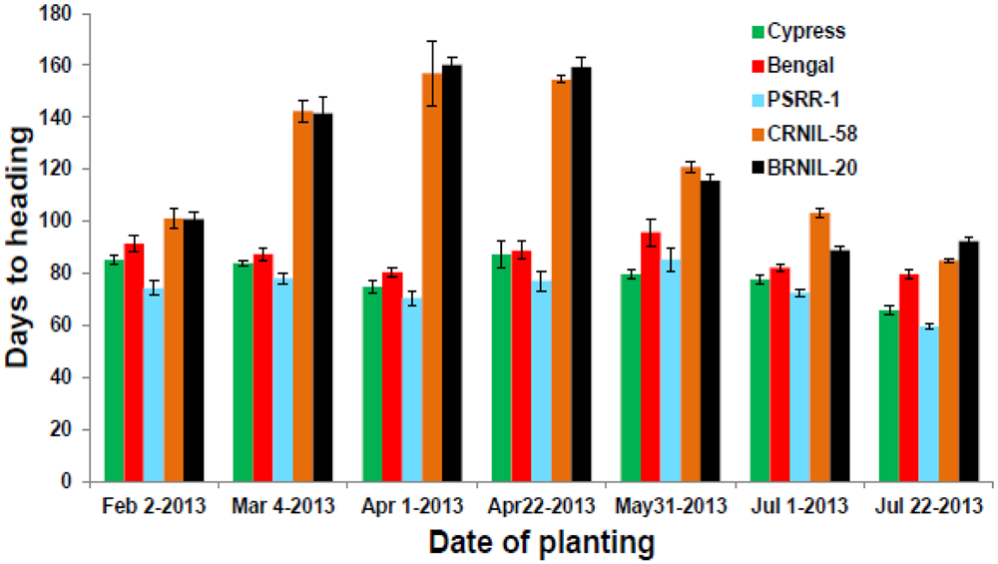
Response of ‘Cypress’, ‘Bengal’, ‘PSRR-1’, BRNIL-20, and CRNIL-58 to photoperiod in a greenhouse experiments. Planting of parents and NILs was staggered at different dates between early February and late July to expose the plants to different lengths of photoperiod. Standard deviations are indicated by the error bars.

The staggered planting experiment in greenhouse revealed that DTH was similar on all planting dates for the parents, but both NILs took a much longer time (>140 days) for flowering compared to parents under LD condition (Fig. 2). DTH peaked for plantings done in month of April and then gradually decreased each month, with the lowest in July. The exposure to LD conditions most likely delayed flowering in these NILs. Upon exposure to SD (10 h day length) in greenhouse, flowering could be induced (Supplementary Fig. S6). Flowering response was studied in ‘Bengal’, BRNIL-20, and its F_1_ under LD condition. The F_1_ and NIL did not flower 105 d after planting in greenhouse (Supplementary Fig. S7). Monitoring of heading date indicated that DTH of F_1_s between the RP and their corresponding NIL was extremely late and photosensitive. The *Hd1* alleles behaved in additive manner in cultivated rice background.

### Sequence variation in *Hd1* gene and *Hd3a* promoter

‘Cypress’ and ‘Bengal’ *Hd1* sequences were identical, but the ‘PSRR-1’ allele differed from both cultivars with a 123 bp deletion, a 36 bp insertion, a 2 bp insertion, and several SNPs (Supplementary Fig. S8). The 123 bp deletion in first exon was present in both ‘PSRR-1’ and ‘Nipponbare’. Nipponbare genome sequence was used for comparison because it is not only the reference genome but also a photosensitive rice variety. The 36 bp insertion was present in the CDS of all three parents but not in ‘Nipponbare’. There was a 2 bp insertion in 2^nd^ exon in in ‘Bengal’ and ‘Cypress’ compared with ‘PSRR-1’ and ‘Nipponbare’. Analysis of cDNA and deduced amino acid sequence revealed truncated amino acid sequences in ‘Bengal’ and ‘Cypress’ compared to ‘PSRR-1’ and ‘Nipponbare’. All three parents had B-Box type zinc finger zinc binding domains like ‘Nipponbare’ but a CCT domain, which photo-insensitive cultivars ‘Bengal’ and ‘Cypress’ lack, was present in both ‘PSRR-1’ and ‘Nipponbare’. ‘Bengal’ and ‘Cypress’ sequences are identical for the *Hd3a* promoter region. But there were 18 SNPs and a 12 bp insertion in ‘PSRR-1’ compared to ‘Cypress’ and ‘Bengal’ (Supplementary Fig. S9).

### Segregation of the *Hd1* allele in the NIL F_2_ mapping population

**Figure 3:**
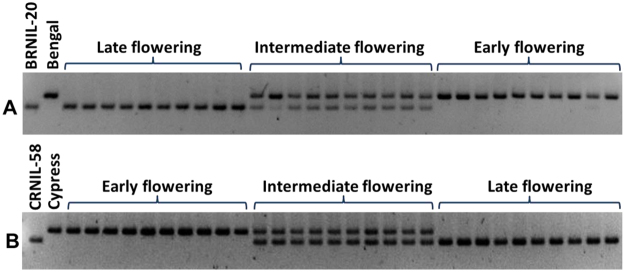
Segregation of *Hd1* alleles in Bengal x BRNIL-20 F_2_ population **(A**) and Cypress x CRNIL-58 F_2_ population (**B**). ‘PSRR-1’ homozygotes were late (>130 days), ‘Cypress’ or ‘Bengal’ homozygotes were early (<90 days), and heterozygotes flowered within 91–130 days. In Cypress x CRNIL-58 F_2_ population, 1020 plants were evaluated for flowering. The *Hd1* genotyping result of a sample of 10 plants from each early, intermediate, and late group in each population is shown.

Using primers flanking the 123 bp deletion in *Hd1*, we confirmed the presence of’PSRR-1’ *Hd1* allele in CRNIL-58 and BRNIL-20. In the case of Cypress x CRNIL-58 cross, the F_2_ population could be classified into three groups: early (<90d), intermediate (91–130 d), and late (>130 d) (Supplementary Fig. S10A) and segregation of these three groups fit into 1:2:1 ratio. Genotyping of the population indicated that all early and late heading plants were homozygous for ‘Cypress’ and ‘PSRR-1’ allele, respectively and the plants with intermediate DTH were heterozygous (Fig. 3B). In the ‘Bengal’ x BRNIL-20 F_2_ population (n = 600), we also confirmed the same pattern of phenotypic segregation (Supplementary Fig. S10B) and genotypic segregation in a pool of 10 randomly selected plants from each early, intermediate, and late flowering group of segregants (Fig. 3A).

### Expression pattern of flowering pathway genes

**Figure 4:**
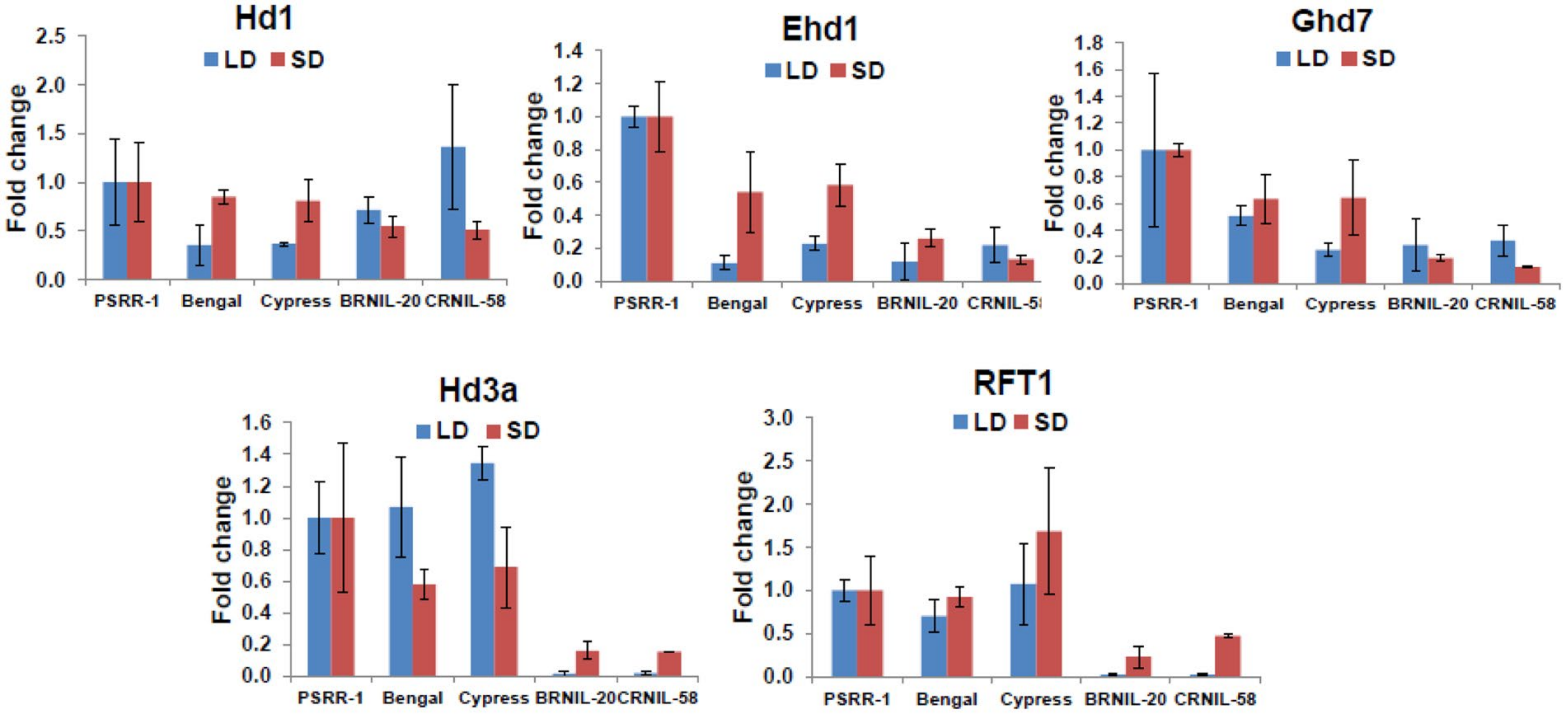
Quantitative RT-PCR analyses of five key flowering pathway genes (*Hd1, Ehd1, Ghd7, Hd3a*, and *RFT1*) in parents and NILs under natural LD and SD conditions. Transcript levels in leaves sampled 55 days after planting were measured in three biological replicates with three technical replications. The mean values of the relative expression levels of genes in BRNIL-20, CRNIL-58, ‘Bengal’, and ‘Cypress’ were compared with ‘PSRR-1’. Standard deviations are indicated by the error bars. The rice *Actin1* gene was used as the internal control for normalization.

An analysis of expression profile of five key flowering genes (Fig. 4) revealed that *Hd1* expression was slightly higher in parents than NILs under SD compared to LD condition. Both NILs and ‘PSRR-1’ had higher accumulation of *Hd1* transcripts compared to both parents under LD. The *Ehd1* transcript level was very high in ‘PSRR-1’, ‘Bengal’, and ‘Cypress’ under SD but its expression was significantly reduced in NILs, ‘Bengal’, and ‘Cypress’ compared to ‘PSRR-1’ under LD. On the other hand, transcript level of *HD3a* and *RFT1* was negligible in NILs under LD while *Ghd7* expression was relatively lower in NILs compared to ‘PSRR-1’ under both SD and LD.

There were three different patterns of expression among the parents and NILs in other flowering pathway genes such as *Ehd2, OsGI, Hd6, ETR2*, and *OsLhy* (Supplementary Fig. S11). There was no difference in transcript level for *OsLhy* under LD, but it was reduced in NILs compared to parents under SD. The *Ehd2* and *Hd6* expression in both NILs was relatively lower than all parents under both SD and LD. While there was not much variation in transcript level of *OsGI* and *ETR2* among all lines under SD, it was relatively higher under LD in both NILs compared to all three parents. The pattern of expression of *ETR2* in LD was exactly opposite to that under SD, i.e. transcript abundance was more in NILs under LD compared to parents, whereas it was more in parents compared to NILs under SD condition.

### Discovery of putative gene interaction

There were three significant digenic QTL interactions in the BR-RIL population (Supplementary Table S2). The effects were negligible for interactions involving *qHD6*^*BR*^ and two other QTLs on chromosome 2. But the most significant one was between *qHD6*^*BR*^ and *qHD7-1*^*BR*^ with LOD score of 24. This interaction was validated using three advanced backcross introgression lines (Supplementary Table S3) and BR-RIL population (Supplementary Table S4) using markers linked to *qHD6*^*BR*^ and *qHD7-1*^*BR*^. Those lines with homozygous PSRR-1 segments in both *qHD6*^*BR*^ and *qHD7-1*^*BR*^ flowered early, but homozygosity for PSRR-1 allele at only *qHD6*^*BR*^ resulted in late flowering and photosensitivity. Comparison of the marker profiles of the late and early flowering RILs revealed that all early flowering RILs were homozygous for the PSRR-1 allele at both RM3431 and RM214 (Supplementary Table S4, Supplementary Fig. S12).

The IL7-3 harboring the *qHD7-1*^*BR*^ flowered significantly earlier than the recurrent parent ‘Bengal’ under both LD and SD conditions (Supplementary Fig. S13), but it was more pronounced under LD. This observation was consistent with the results from QTL analysis that PSRR-1 allele at *qHD7-1*^*BR*^ was responsible for reducing the DTH. The F_1_ between the IL7-3 and BRNIL-20 was intermediate in flowering and the segregation of early, intermediate, and late flowering plants in the F_2_ population fit into 5:8:3 ratio (Supplementary Fig. S14). Based on digenic interaction model (Supplementary Fig. S15), early flowering occurred when both genes were in homozygous condition for the ‘PSRR-1’ alleles. The gene on chromosome 7 putatively interacting with *Hd1* was having no effect in homozygous recessive or heterozygous condition. Since *Ghd7* was present in the introgressed segment of IL7-3, we initially hypothesized *Ghd7* as the candidate interacting with *Hd1*. Expression of *Hd1*, *Hd3a*, and *Ghd7* under SD was reduced in IL7-3 compared with ‘Bengal’ (Supplementary Fig. S16). To confirm the interaction hypothesis, an early flowering segregant (#229) homozygous for ‘PSRR-1’ allele of both *Hd1* and *Ghd7* was selected from the F_2_ population of the cross BRNIL-20 × IL7-3. If *Ghd7* is the candidate, all F_3_ progenies were expected to flower early. However, we noticed that 9 plants were extremely late and 21 plants were either early or intermediate flowering type suggesting single gene segregation. Both early and late flowering plants were homozygous for the ‘PSRR-1’ *Ghd7* allele (Supplementary Fig. S17). The physical location of *Ghd7* was at 9.2 Mb, whereas the QTL peak was near RM214 located around 12.7 Mb position. Expression analysis of both *Hd1* and *Hd3a* in F_3_ progenies of #229 indicated that even though the expression of *Hd1* in both early and late plants was the same, higher expression of *Hd3a* was observed in early heading plants compared with late ones (Supplementary Fig. S18).

## Discussion

Natural genetic variation has been exploited to decipher the genetic basis of flowering in response to photoperiod^37^. In this study, we used both RIL^34^ and IL^35^ populations developed from crosses involving cultivated and weedy rice, which allowed assessment of the magnitude of the QTL effects on phenotype as well as discovery of a genetic interaction. The increased power of the IL population to detect novel and more QTLs compared to RIL populations was due to reduced genetic noise resulting from the segregation of fewer QTLs at the same time. It is particularly valuable under circumstances when large effect QTLs are masked by complex genetic interactions. In addition to the validation of several previously cloned genes^7,38,39^, few novel QTLs were also discovered in this study (Fig. 1). A genetic background effect was clearly evident from the QTL results in both populations. For example, the QTL *qHD7-1*^*BR*^ with largest effect in the BR-RIL population was not detected in the CR-RIL population. Similarly, the QTL corresponding to the largest effect *qHD3*^*CR*^ in the BR-RIL population had negligible effect. Development of ILs of ‘PSRR-1’ in the ‘Cypress’ background and comparison with the BR-ILs will be helpful to investigate the reasons for such discrepancies.

Development of early maturing rice varieties is an important breeding objective. In this study, we discovered that weedy rice alleles could be useful to shorten the maturity duration (For example, *qHD7*^*CR*^ in CR population, *qHD1*^*BR*^, *qHD2-2*^*BR*^, *qHD7-1*^*BR*^, *qHD7-2*^*BR*^, and *qHD11*^*BR*^ in BR population). Particularly, the transfer of *qHD7-1* from ‘PSRR-1’ to several genetic backgrounds could be exploited in breeding early maturing varieties for US rice growing environments.

Results from previous studies on genetic interaction in weedy rice^40,41^ were different from the present study demonstrating the variability in weedy rice populations and complex genetic interactions involving several heading date genes and their variants. Weedy rice alleles at three QTLs - *Se7*.*1, Se7*.*2*, and *Se8* inhibited flowering^40^. In the other study^41^, a nonfunctional *Hd1* crop allele and a weed allele of a QTL *qHD7S* increased the heading date, whereas a weedy *Hd1* allele with crop allele at *qHD7S* locus resulted in early flowering. But we demonstrated that early flowering was due to the combination of the weedy alleles of *qHD7-1* and *Hd1* in a cultivated rice background. Most of these studies used photosensitive parents, which is in sharp contrast to the use of photo-insensitive parents in this study.

The weedy rice accession and both rice cultivars used in this study were all day-neutral. However, ‘Nipponbare’ and ‘Kasalath’, which were used to clone *Hd1*^7^, were photosensitive and weakly photosensitive, respectively. Strong photosensitivity response in BR and CR hybrids provided the first evidence for the genetic interaction. The *Hd1* allele of ‘PSRR-1’ was functional like ‘Nipponbare’ due to the presence of CCT domain. Using the NILs developed in two genetic backgrounds, we demonstrated that the *Hd1* allele of ‘PSRR-1’ was responsible for late flowering and photosensitive response. Although the effect of *Hd1* was additive in NILs, strong photosensitivity and extremely late flowering observed in BR and CR hybrids could be due to the segregation of other genes influencing this trait. Since both RIL populations segregating for photoperiodic response involved non-contrasting parents, we hypothesized that gene interaction involving *Hd1* was responsible for the photo-insensitivity in ‘PSRR-1’ and the gene interacting with *Hd1* should be in homozygous condition.

Our results demonstrated the role of *Hd1* and its genetic interaction in regulating flowering and photoperiodic response. We ruled out the *Hd3a* promoter sequence variation regulating flowering^27^ because there was no difference in transcript level of *Hd3a* among the parents irrespective of day length variation (Fig. 4). Although the transcript level of *Hd1* in both NILs was adequate under LD, the expression of both florigen genes, *Hd3a* and *RFT*, was negligible (Fig. 4). As the transcript levels of *Ehd1* and *Ghd7* in both NILs were comparable with their respective recurrent parents under LD, the introgressed *Hd1* may not be regulating transcription of these genes. However, introgression of *qHD7-1* and *Hd1* from ‘PSRR-1’ in’Bengal’ background resulted in early flowering under LD due to upregulation of the *Hd3a* gene (Supplementary Fig. S18). The observation that the early flowering F_2_ individuals from the cross between *Hd1* NIL (BRNIL-20) and IL7-3 harboring *qHD7-1*^*BR*^ were homozygous for *Hd1* allele of ‘PSRR-1’, provided further evidence for genetic interaction between *Hd1* and an unknown factor in the *qHD7-1*^*BR*^ region.

The interaction of *Hd1* with *Ghd7* was previously reported to regulate photoperiodic flowering^31,32^. The flowering induction or suppression activity of *Hd1* under LD was dependent on the *Ghd7* allelic status^31^. Both studies^31,32^ demonstrated that the physical interaction between the CCT domain of *Ghd7* and the transcription activation domain of *Hd1* led to suppression of expression of *Ehd1* and florigen genes *Hd3a/RFT1* under LD condition resulting in late flowering. Since *Ghd7* was located in the *qHD7-1*^*BR*^ region, it was necessary to determine if *Ghd7* or a new gene is interacting with *Hd1* leading to early flowering response. We summarized below the evidences to support the involvement of a new gene other than *Ghd7* in this newly discovered genetic interaction, which is an important finding of this study. It was hypothesized earlier that the gene involved in the interaction with *Hd1* should be in homozygous condition. The phenotypic segregation of the F_2_ population into 5 early:8 intermediate:3 late ratio in the cross between BRNIL-20 and IL7-3 (Supplementary Fig. S15) proved the above hypothesis. The occurrence of late flowering plants that were homozygous for both *Hd1* and *Ghd7* alleles of PSRR-1 also eliminates the possible involvement of *Ghd7*. We further selected an early flowering F_2_ plant #229 which was homozygous for the weedy *Hd1* and *Ghd7* alleles as well as for the markers (RM7121, *Rc*, RM214, and RM5793) located in the introgressed *qHD7-1*^*BR*^ region. Instead of uniform early flowering response, which is expected if *Ghd7* is involved in interaction, the F_3_ progenies of the plant #229 segregated for flowering implying involvement of a new gene. Since the plant #229 still retained a large introgressed segment of PSRR-1 flanking *Ghd7*, it is highly unlikely that recombination within the gene resulted in chimeric *Ghd7* in such a small population (n = 282). Despite similar transcript level of *Hd1* in both early and late F_3_ segregants of plant #229, expression of *Hd3a* and *Ghd7* was higher in the former compared to the later under LD (Supplementary Fig. S18). The differential transcript level of *Ghd7* and *Hd3a* in both early and late group of plants may be attributed to the new gene. Another evidence against the involvement of *Ghd7* was based on the fact that *Ghd7* (9.15 Mb position) is physically located far away from RM214 (12.78 Mb position), which was closely linked to the *qHD7-1*^*BR*^ (Fig. 1) and was demonstrated to interact with RM3431 (closely linked to *Hd1*) in the BR-RIL population (Supplementary Table S4).

Although the physical interaction between *Ghd7* and *Hd1* was responsible for extreme late flowering under LD condition, Nemoto *et al*. (2016)^32^ did not rule out involvement of other genes or other mechanisms for the photoperiod-dependent reversal of *Hd1* function. Their study^32^ provided many clues for involvement of unidentified genes other than *Ghd7* in suppression of *Hd1* supporting our conclusion in this study. For example, *Hd1* overexpressing Kita-ake with a nonfunctional *Ghd7* delayed flowering under both SD and LD. But in our study, weedy *Hd1* allele and cultivar *Ghd7* allele were present in extreme late flowering BRNIL-20 and CRNIL-58. On the contrary, the IL7-3 with a nonfunctional *Hd1* allele and the weedy rice segment harboring weedy allele of *Ghd7* flowered significantly earlier than the recurrent parent under both SD and LD (Supplementary Fig. S13). It was also speculated that the interaction among the flowering genes was dependent on developmental stages of the plant^32^ and *Hd1* may be interacting with gene (s) other than *Ghd7* at the vegetative stage of rice. Considering the above facts, it is highly likey that a new gene, possibly the one hypothesized in our study, may be involved in interaction with *Hd1* for reversing its role as a transcriptional activator leading to promotion of flowering under LD condition. However, it remains to be determined if the same gene in IL7-3 is responsible for both early flowering and interaction with *Hd1*, thus warranting further investigation including cloning of *qHD7-1*^*BR*^.

**Figure 5:**
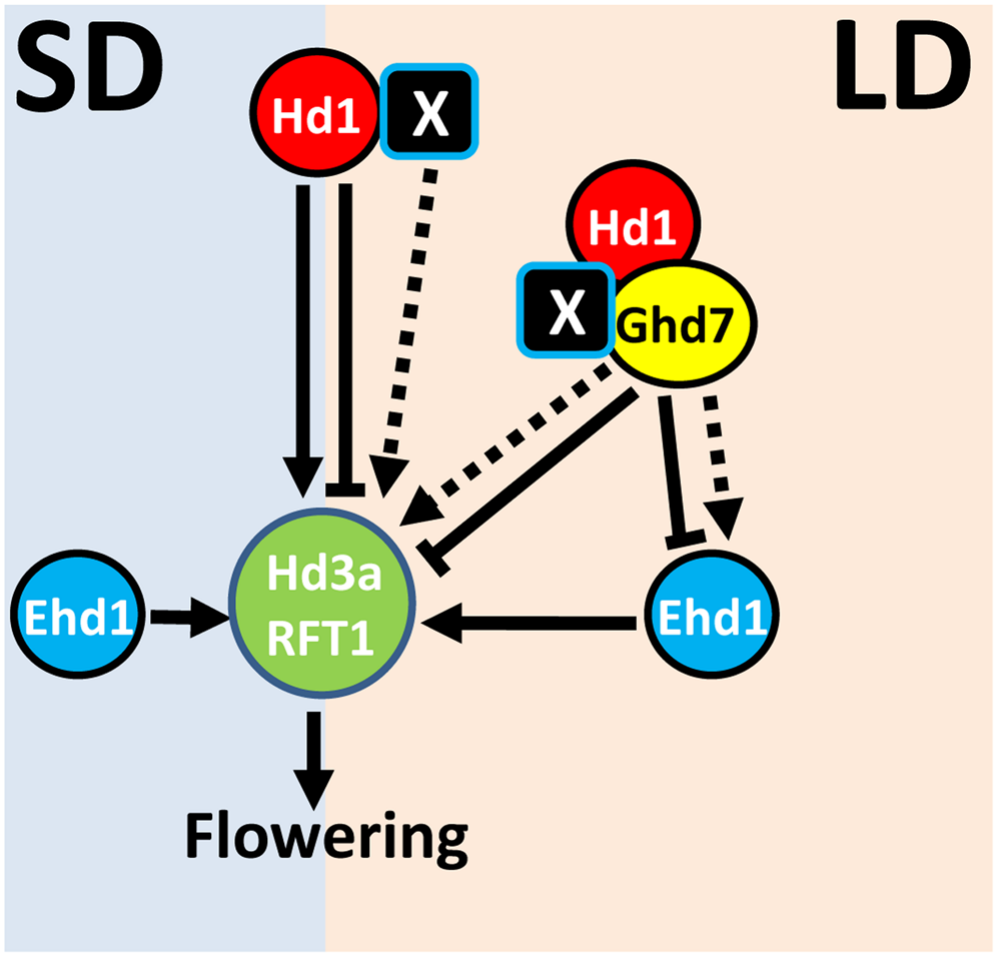
A simplified model for induction or repression of flowering in rice under LD condition. The new gene (X) is proposed to restore of the expression of *Hd3a/RFT1* leading to initiation of flowering through direct interaction with Hd1 or Hd1/Ghd7 complex or through activation of *Ehd1*. Transcription activation and repression of major flowering genes based on current literature are indicated by solid arrows and flat arrows, respectively. The proposed mode of regulation of flowering by the new gene (X) is marked with dotted arrows. SD: short-day, LD: long-day.

Our results suggest the new gene as a missing link between *Hd1* and florigen genes and may be functioning downstream of *Hd1* involving a post transcriptional mechanism. We propose to integrate the role of this new gene in the flowering pathway in following ways (Fig. 5): (a) The new gene may interact with the *Hd1* to regulate the florigen genes, (b) the new gene product may physically interact with Ghd7/Hd1 complex to directly regulate florigen genes or through *Ehd1* since multistep regulation of the downstream genes is possible. In conclusion, our study not only demonstrates unlocking of the hidden genetic diversity underlying the flowering time variation in response to photoperiod in weedy rice, but also emphasizes the need to discover novel loci and their genetic interactions for rice improvement.

## Materials and Methods

### Plant materials

Two RIL populations were developed from the cross combinations, Bengal x PSRR-1 and Cypress x PSRR-1. The Bengal × PSRR-1 RIL population (named as BR) was composed of 198 individuals in the F_7:8_ generation, whereas the ‘Cypress × PSRR-1 population (named as CR) included 174 RILs in the F_8:9_ generation^34^. ‘Bengal’ and ‘Cypress’ are high yielding rice cultivars developed by the Louisiana Agricultural Experiment Station^42,43^. PSRR-1 is a weedy rice accession with light green pubescent leaves, vigorous growth habit, straw-hulled medium grain, open panicles, high seed shattering, and intense seed dormancy. It was purified by selfing for two generations from the seeds collected from the Rice Research Station of the LSU Agricultural Center located at Crowley, Louisiana. Weedy rice is a conspecific form of cultivated rice (*Oryza sativa* L.), which has been a major constraint for rice production in the US and other parts of the world.

A population of 74 homozygous introgression lines (ILs) covering the entire PSRR-1 genome in ‘Bengal’ background was developed by three rounds of backcrossing followed by two generations of selfing^35^ (Supplementary Fig. S19). Marker assisted selection was employed in each generation to speed up the development of ILs with fewer donor segments and the IL population was in the BC_3_F_3_ generation. BRNIL-20 was a photosensitive NIL of PSRR-1 in ‘Bengal’ background developed by additional backcrossing of one of the photosensitive IL^35^. CRNIL-58 was another photosensitive NIL of ‘PSRR-1’ in ‘Cypress’ background which was developed in the same manner without employing marker-assisted selection. It was identified in the BC_3_F_3_ generation. Both photosensitive NILs contained a single ‘PSRR-1’ introgression in chromosome 6 based on genotyping using polymorphic simple sequence repeat (SSR) markers^34^.

Two F_2_ populations were developed by crossing BRNIL-20 and CRNIL-58 to their respective recurrent parents to determine if *Hd1* is responsible for heading date variation. IL7-3 was an early flowering IL of ‘PSRR-1’ in ‘Bengal’ background with only one introgressed segment harboring the *qHD7-1*^*BR*^ region^35^. An F_2_ population was developed from the cross between BRNIL-20 and IL7-3 and an F_2_ plant (#229) homozygous for PSRR-1 *Hd1* and *Ghd7* allele was selected and was evaluated in the F_3_ generation for the heading date in greenhouse.

### Phenotyping and genotyping

The parents and both RIL populations were grown at the Central Research Station of the LSU Agricultural Center in Baton Rouge, LA (30°20′51″N, 91°10′14″W). Planting was done in the middle of April to ensure exposure to natural long-day conditions. Each line was sown in a 2-m row of 20 plants with a row spacing of 20 cm. Standard cultural practices were followed. The heading dates in both mapping populations were recorded on five randomly selected plants from each line. ‘Days-to-heading’ was defined as the number of days from seeding to the first panicle emergence in each plant. Mean temperature and day length between planting and harvesting ranged from 21 °C to 29 °C and 12 to 14 hrs, respectively. Average day length data compiled over four years (2009, 2011, and 2013–2015) indicated that day length was longest (13–14 h) in May and June (Supplementary Table S5).

The IL population and parents were evaluated at the same location following the same planting plan and cultural practices as described above. The photosensitive NILs, parents, and F_2_ populations from the crosses, Bengal x BRNIL-20 (n = 600) and Cypress x CRNIL-58 (n = 1020), were grown in field condition. The individual plants were classified as early, intermediate, and late when flowering occurred <90 days, 91–130 days, and >130 days, respectively. A sample of 200 F_2_ individuals of the Cypress x CRNIL-58 cross was genotyped using the *Hd1* deletion primers (Supplementary Table S6), whereas 10 randomly selected plants from each early, intermediate, and late flowering group were used for genotyping in the F_2_ population involving BRNIL-20. The IL7-3 was evaluated under both SD and LD conditions by growing them in late July and mid-April, respectively. To investigate the genetic interaction involving the *Hd1* locus, the F_2_ population from the cross, BRNIL-20 × IL7-3 (n = 282), was evaluated for heading date and genotyped for *Hd1, Ghd7*, and other marker loci in the introgressed PSRR-1 region in BRNIL-20 and IL7-3. Unless otherwise specified, all heading date evaluations were conducted under long-day conditions.

The response to photoperiod in Cypress, Bengal, PSRR-1, BRNIL-20, and CRNIL-58 was evaluated in a greenhouse experiment. Planting of parents and NILs was staggered at different dates between early February and late July to expose the plants to different lengths of photoperiod. Each genotype was replicated five times with one plant per pot and same cultural practices such as application of fertilizer and pesticides were followed for all genotypes. Plants were placed on the same bench without any movement inside the greenhouse throughout the growing period. Days to heading was recorded on five plants and mean values were used for analysis.

### QTL mapping and statistical analysis

Linkage maps developed earlier for both RIL populations^34^ were used for QTL mapping. The linkage maps of BR and CR RIL populations consisted of 212 and 189 SSR markers with total map lengths of 1410 and 1574 cM, respectively. QTL Cartographer Version 2.5^44^ was used for QTL analysis following a composite interval mapping (CIM) procedure. Logarithms of odds (LOD) threshold values for CIM were determined based on 1000 permutations to declare significant QTLs at P < 0.01. For BR and CR populations, these LOD values were 3.73 and 3.36, respectively. The QTLs identified at LOD 2.5 were included as suggestive QTLs. CIM was performed by using the standard model (model 6) in the backward regression method, which included the selection of 20 markers as cofactors with a window size of 10 cM to identify QTLs with 1 LOD confidence interval. The total phenotypic variation explained by all putative QTLs was estimated by fitting a model in the multiple interval mapping procedure of QTL Cartographer. Interactions among the identified QTLs were detected using the multiple interval mapping method in the QTL Cartographer. The nomenclature of QTLs was done by adding a superscript of ‘BR’ or ‘CR’ after the QTL to indicate a QTL identified in BR or CR populations, respectively.

The presence of QTL in the IL mapping population was inferred when there was a significant difference between the mean of an IL and the recurrent parent ‘Bengal’ using the Dunnett’s test. Analysis of variance and Dunnett’s test were performed in Statistical Analysis System (SAS) software version 9.4 for Windows^45^. Substitution mapping^36^ was followed to narrow down the QTL region using ILs with overlapping chromosome segments. The additive effect of each QTL was estimated as half the difference between trait mean of the IL and the trait mean of the recurrent parent^46^. All histograms were constructed in Microsoft Excel 2010.

### Sequencing of *Hd1* gene and *Hd3a* promoter

The entire genomic and cDNA sequences of *Hd1* of ‘PSRR-1’, ‘Bengal’, and ‘Cypress’ were amplified from the genomic DNA and cDNA using Phusion High Fidelity DNA Polymerase (New England Biolab, MA) with primers listed in Supplementary Table S6. Similarly, the *Hd3a* promoter and 5′ UTR region upstream of ATG (~1975 bp) was amplified from genomic DNA of ‘PSRR-1’, ‘Bengal’, and ‘Cypress’. Primers (Supplementary Table S6) were designed based on the available reference genome sequences of the Nipponbare in the rice genome annotation database (http://rice.plantbiology.msu.edu/). The gel purified PCR products were first cloned into the pGEM-T Easy vector system I (Promega Corp., WI) and three independent products were sequenced at the Genomic Facility of Louisiana State University. Genomic DNA, CDS, and deduced protein sequences were aligned using the MegAlign module of the Lasergene genomics suite 14.0 (DNASTAR, Madison, WI). The 123 bp deletion of *Hd1* genomic sequence was targeted to distinguish the *Hd1* alleles via PCR using a pair of primers under the following thermocycler profile: 95 °C for 5 min; 35 cycles of 94 °C for 45 s, 60 °C for 45 s and 72 °C for 1 min; and a final extension at 72 °C for 5 min.

The genome sequence of ‘Bengal’ and ‘PSRR-1’ available in our laboratory was used to develop SNP markers for the *Ghd7* gene. The *Ghd7* alleles of ‘PSRR-1’ and ‘Bengal’ were amplified using primers Ghd7-F/Ghd7-RR-R and Ghd7-F/Ghd7-BN-R, respectively, using the following thermocycler profile: initial denaturation at 94 °C, 3 min; 35 cycles of 94 °C for 30 s, 60–65 °C for 30 s and 72 °C for 45 s; and a final extension at 72 °C for 5 min.

### Quantitative real-time polymerase chain reaction (qRT-PCR) analysis

Seeds of parents (Cypress, PSRR-1, and Bengal), and NILs (BRNIL-20 and CRNIL-58) were sown in mid-April and mid-July for exposure to natural LD condition and SD conditions, respectively. Top leaves were sampled for gene expression analysis 55 days after sowing. Total RNA was isolated using TRIZOL reagent (Invitrogen, Carlsbad, CA, USA), followed by treatment with TURBO^TM^ DNA-free *DNase* (Invitrogen, Carlsbad, CA, USA) to remove possible genomic DNA contamination. Quality of total RNA was checked in a 1.2% formamide-denaturing agarose gel, and quantification was done using a ND-1000 spectrophotometer (NanoDrop Technologies, Inc., Wilmington, USA). First-strand cDNAs were synthesized using the iScript cDNA Synthesis Kit (Bio-Rad, Hercules, USA) in a reaction volume of 20 µL. Gene specific primers for qRT-PCR (Supplementary Table S6) were designed for known flowering pathway genes using Primer3Web (version4.0.0) software (http://bioinfo.ut.ee/primer3). The expression level of these genes was determined using a MyiQ BioRad Single Color Real-time PCR Detection System following the protocol described earlier^47^. Each 10 µl of PCR sample contained 5 µl of SYBR Green mix (Bio-Rad, Hercules, USA), diluted cDNA, and 0.4 µM of forward and reverse gene specific primers. The expression of each gene in different RNA samples was normalized with the expression of an internal control gene, rice *Actin1* (LOC_Os05g36290.1). Melt curve analysis was performed to check the specificity of the amplified product and fold changes in mRNA expression of each gene in different genotypes compared to PSRR-1 was calculated^48^. Each CT (cycle threshold) value represented the average of three biological replicates with three technical replicates.

## Electronic supplementary material

Supplementary Figures and Tables
