# Supplementary material for: Genetic interaction involving photoperiod-responsive Hd1 promotes early flowering under long-day conditions in rice
Source: Sci Rep. 2018 Feb 1;8:2081. doi: 10.1038/s41598-018-20324-1 (PMC5794782; doi:10.1038/s41598-018-20324-1)
Supplement: Supplementary file 1 — Supplementary Figures and Tables [file 41598_2018_20324_MOESM1_ESM.pdf]

Supplementary Materials for

Genetic interaction involving photoperiod-responsive *Hd1* promotes early flowering under long-day conditions in rice

Prasanta K. Subudhi†, Teresa De Leon, Ronald Tapia, Chenglin Chai, Ratna Karan, John Ontoy, and Pradeep K. Singh

†Corresponding author. Email: psubudhi@agcenter.lsu.edu

Supplementary Figures S1 to S19

Supplementary Tables S1 to S6

Supplementary Figure Legends

Figure S1. Frequency distribution for days to heading in the BR-RIL population (A) and CR-RIL population (B) in the field. Phenotypic values of both parents are indicated by arrows.

Figure S2. Genome-wide QTL profiles of heading date in the BR-RIL population. Top panel represents the QTL profile while the bottom panel is the additive effect of the 'Bengal' allele. Threshold LOD value of 2.5 was used for identification of QTLs.

Figure S3. Genome-wide QTL profiles of heading date in the CR-RIL population. Top panel represents the QTL profile while the bottom panel is the additive effect of the 'Cypress' allele. Threshold LOD value of 2.5 was used for identification of QTLs.

Figure S4. Frequency distribution of days to heading in PSRR-1 IL population developed in the 'Bengal' background[35]. Phenotypic values of both parents are indicated by arrows.

Figure S5. Graphical genotyping of the photosensitive BRNIL-20 and CRNIL-58 with a single PSRR-1introgressed segment from chromosome 6. Chromosome maps were from the RIL linkage maps[34]. The peak position of QTL was at RM3431 (8.74 Mb) and RM8225 (9.31 Mb) in BR and CR-RIL populations, respectively. The physical map location of markers and flowering genes (Mb position based on reference genome) are given in parentheses.

Figure S6. Flowering in CRNIL-58 after exposure to 10 hr photoperiod (A) but no sign of flowering under natural long-day conditions (B). Planting date was April 18, 2012.

Figure S7. Flowering response to natural long-day conditions in 'Bengal' (A), BRNIL-20 (B), and its F_1 (C) 105 days after planting (planting date: April 18, 2012). The heading date of F_1 plants was intermediate, whereas the NIL was very late.

Figure S8. Alignment of the *Hd1* genomic (A), cDNA (B), predicted amino acid sequences (C), and domain analysis (D) of 'Nipponbare', 'Bengal', 'Cypress', and 'PSRR-1'.

Figure S9. Alignment of *Hd3a* promoter and 5' UTR regions of 'Bengal', 'Cypress', and 'PSRR-1'.

Figure S10. Frequency distribution of plants in F_2 populations from the crosses, Cypress x CRNIL-58 (n=1020) (A) and Bengal x BRNIL-20 (n=600) (B) for heading date in a field experiment in 2014. The F_2 plants flowering in ≤90, 91-130, and >130 days were classified as early (E), intermediate (I), and late (L), respectively. The segregation ratio fit into 1:2:1 (Chisquare values were 0.564 and 1.153 for the Cypress x CRNIL-58 and Bengal x BRNIL-20 F_2 populations, respectively.

Figure S11. Quantitative RT-PCR analysis of flowering pathway genes (*Ehd2, Hd6, OsGI, ETR2,* and *OsLhy*) in parents and NILs under natural LD and SD conditions. Transcript levels in leaves sampled 55 d after planting were measured in three biological replicates with three technical replications. The mean values of the relative expression levels of genes in BRNIL-20, CRNIL-58, 'Bengal', and 'Cypress' were compared with 'PSRR-1' and standard errors were indicated by the error bars. The rice *Actin1* gene was used as the internal control for normalization.

Figure S12. Validation of the two locus interaction for photoperiodic flowering in the BR-RIL population. The QTLs $qHD6^{BR}$ and $qHD7\text{-}1^{BR}$ were represented by their nearest markers

RM3431 and RM214, respectively. The labels AA and aa on horizontal axis represented 'PSRR' homozygotes and 'Bengal' homozygotes, respectively, for marker RM3431. BB and bb represent 'PSRR' homozygotes and 'Bengal' homozygotes, respectively, for marker RM214.

Figure S13. Days to heading in IL7-3, 'Bengal', and 'PSRR-1' under long-day (A) and short-day (B) conditions in greenhouse experiments. Planting was done on 16th April, 2015 and July 22, 2015, to expose the plants to long-day and short-day conditions, respectively. One-way analysis of variance was used to determine differences between lines at $P<0.01$. Values are mean ± standard error of five plants per genotype. Graphical genotype of the IL7-3, an IL of PSRR-1 with single introgression indicated by solid black bar on chromosome (C). *Ghd7* (9.15 Mb position on the reference rice genome) is located in the introgressed region of the IL7-3 based on the physical map location. Chromosome map was from the RIL linkage map[34].

Figure S14. Frequency distribution of days to heading in the F_2 population (n=282) derived from the cross BRNIL-20 x IL7-3. Planting was done in mid-April 2014 to expose the plants to natural long-day condition. The F_1 between BRNIL-20 and IL7-3 flowered in 118 days. Days to heading in IL7-3 was significantly different from 'Bengal' due to substitution of the 'Bengal' alleles with 'PSRR-1' allele for the $qHD7\text{-}1^{BR}$. The F_2 plants flowering in ≤90, 91-130, and >130 days were classified as early (E), intermediate (I), and late (L).

Figure S15. Model for genetic interaction between *Hd1* and an unknown locus on chromosome 7 near RM214 for flowering response. *Hd1* alleles of 'PSRR-1' and 'Bengal' were represented as A and a, respectively. The alleles of 'PSRR-1' and 'Bengal' for the

unknown gene corresponding to *qHD7-1*^{BR} on chromosome 7 were represented as B and b, respectively. Both loci in homozygous condition for 'PSRR' allele were required for photo-insensitivity. The early, intermediate, and late flowering plants in the F_2 population from the cross BRNIL-20 x BRIL7-3 segregated in 5:8:3 ratio (n=282). All late flowering plants were homozygous for 'PSRR-1' *Hd1* allele. Frequency distribution of the F_2 population from this cross was shown in Fig. S14.

Figure S16. Comparative measurements of gene expression of flowering genes (*Hd1, Hd3a,* and *Ghd7)* in leaf tissues of 30 day old seedlings of 'PSRR' and IL7-3, compared to 'Bengal' under short-day conditions. The rice *Actin1* gene was used as the internal control for normalization. The mean values were based on the average of three biological replicates per genotype calculated using relative quantification method[48].

Figure S17. Segregation for the *Ghd7* SNP alleles in parents and 18 early and late flowering F_3 progenies derived from the early flowering F_2 plant #229, which was selected from the cross BRNIL-20 x IL7-3. All early and late flowering plants had 'PSRR-1' *Ghd7* allele. Specific targeted alleles were amplified using primers Ghd7-F/Ghd7-RR and Ghd7-F/Ghd7-BN for 'PSRR-1' (RR) and 'Bengal' (Bng) genotypes, respectively.

Figure S18. Comparative measurements of gene expression of flowering genes (*Hd1, Hd3a,* and *Ghd7*) in leaf tissues of 30 day old early and late flowering F_3 plants from the F_2 plant #229 compared to 'Bengal' under long-day condition. The plant #229 was selected from the F_2 population from the cross BRNIL-20 x IL7-3. The rice *Actin1* gene was used as the internal control for normalization. The mean values were based on the average of three biological replicates per genotype calculated using relative quantification method[48].

Figure S19. A schematic diagram of the development of the introgression lines (ILs) of the weedy rice accession 'PSRR-1' in the cultivated rice 'Bengal' background[35]. MAS: Marker assisted selection.

Supplementary Tables

Table S1. Quantitative trait loci, additive effects, and direction of phenotypic effect for heading date derived from the evaluation of the ILs and the recurrent parent (RP) 'Bengal'. The presence of QTLs was inferred when there was significant difference between the means of each IL and the recurrent parent using Dunnett's test. All ILs significantly different from the recurrent parent at $p < 0.05$ were listed. These data were used to narrow down the QTL regions using substitution mapping. Direction of phenotypic effect (DPE) was denoted by B and R, which indicates either the 'Bengal' and 'PSRR-1' allele increasing the trait values, respectively. The mean days to heading for 'Bengal' and 'PSRR-1' were 76.1 ± 0.4 d and 87.9 ± 1.2 d, respectively. Additive effect (AE) was calculated as follows: Additive effect = (mean of IL- mean of Bengal/2.

Table S2. Epistatic QTLs for heading date identified in RIL populations developed from the Bengal x PSRR-1 and Cypress x PSRR-1 crosses.

Table S3. Identification of putative genetic interactions between a chromosome 6 segment and a chromosome 7 segment for photoperiodic flowering using introgression lines from the BR cross. These three introgression lines had only three substituted 'PSRR-1' segments of which the chromosomes 6 and 7 segments attained homozygosity and chromosome 10

segment (RM1146-RM3451-RM228-RM333) was heterozygous. A and B represent 'Bengal' homozygotes and 'PSRR-1' homozygotes, respectively.

Table S4. Validation of genetic interactions between *qHD6*^{BR} and *qHD7-1*^{BR} involved in photoperiodic flowering in the BR-RIL population. The genotypic profiles of highly photosensitive late flowering RILs and very early flowering RILs were compared for both chromosomal regions harboring the QTLs. The *qHD6*^{BR} corresponded to the *Hd1* locus. The nearest markers for *qHD6*^{BR} and *qHD7-1*^{BR} were RM3431 and RM214, respectively. A and B represent 'Bengal' homozygotes and 'PSRR-1' homozygotes, respectively. *Rc* is a morphological marker for red pericarp.

Table S5. Average day length (A) and temperature (B) in Baton Rouge, Louisiana, USA in 2009, 2011, 2013, 1014, and 2015.

Table S6. List of primers used in this study.

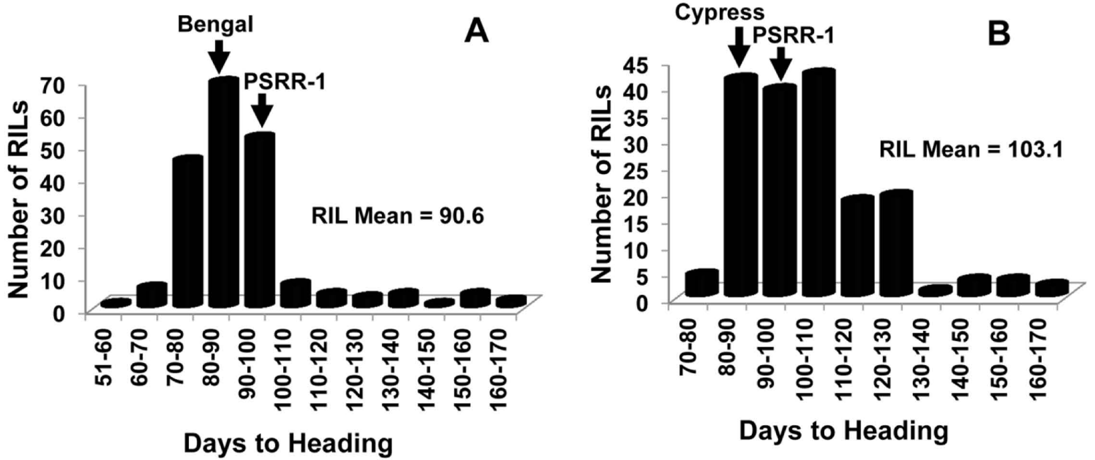

Supplementary Figure S1 Frequency distribution for days to heading in the BR-RIL population[34] (A) and CR-RIL population[34] (B) in the field. Phenotypic values of both parents are indicated by arrows. .

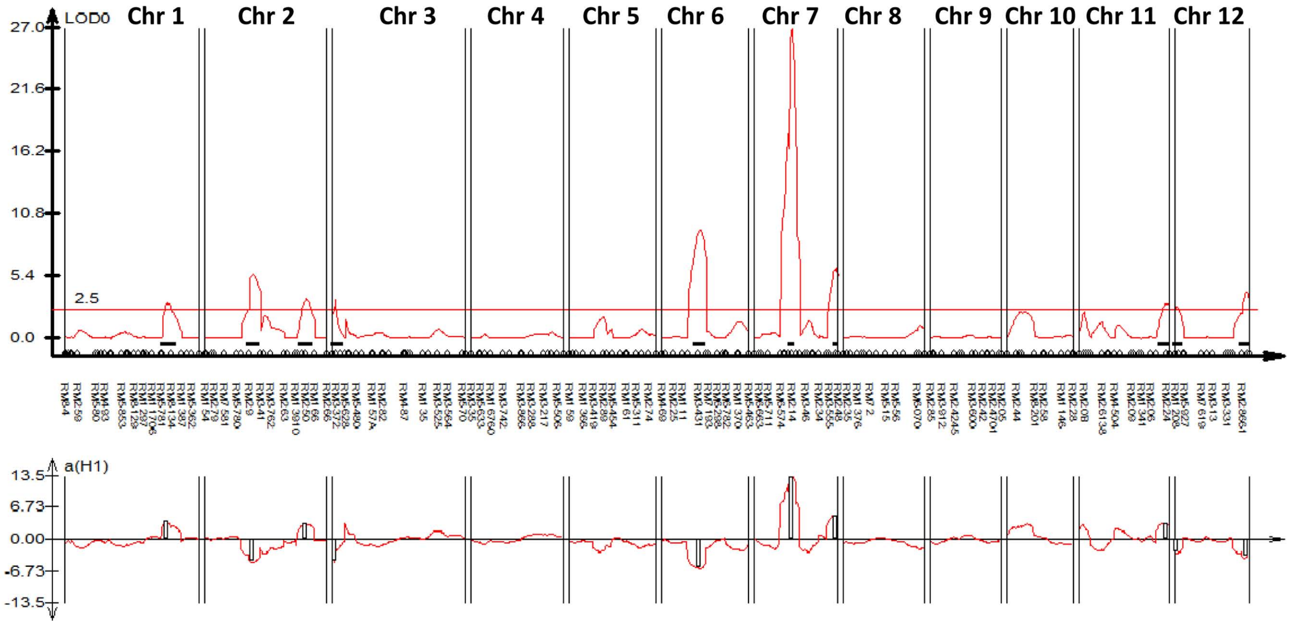

Supplementary Figure S2 Genome-wide QTL profiles of heading date in the BR-RIL population. Top panel represents the QTL profile while the bottom panel is the additive effect of the ‘Bengal’ allele. Threshold LOD value of 2.5 was used for identification of QTLs.

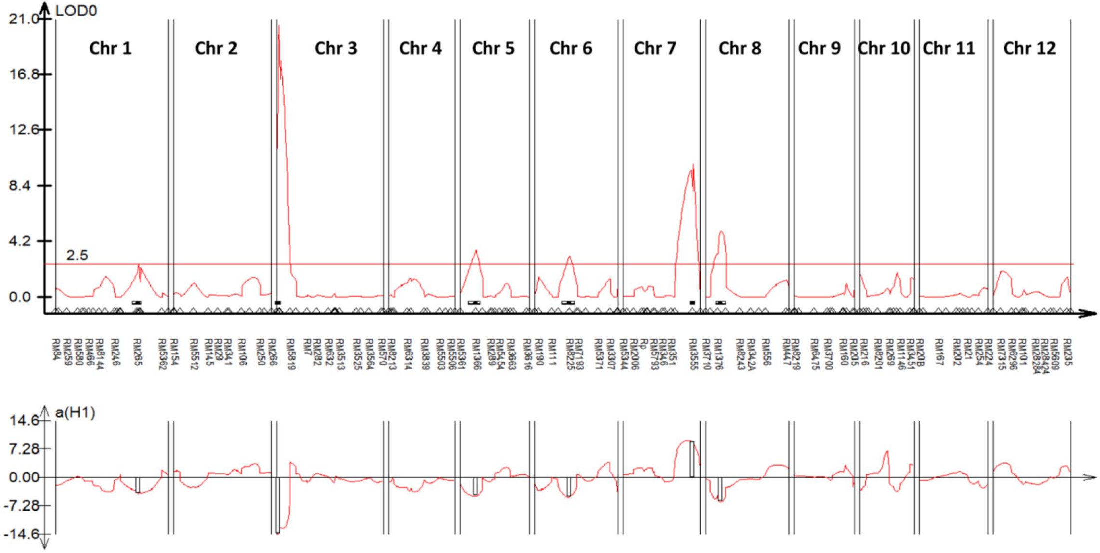

Supplementary Figure S3 Genome-wide QTL profiles of heading date in the CR-RIL population. Top panel represents the QTL profile while the bottom panel is the additive effect of the 'Cypress' allele. Threshold LOD value of 2.5 was used for identification of QTLs.

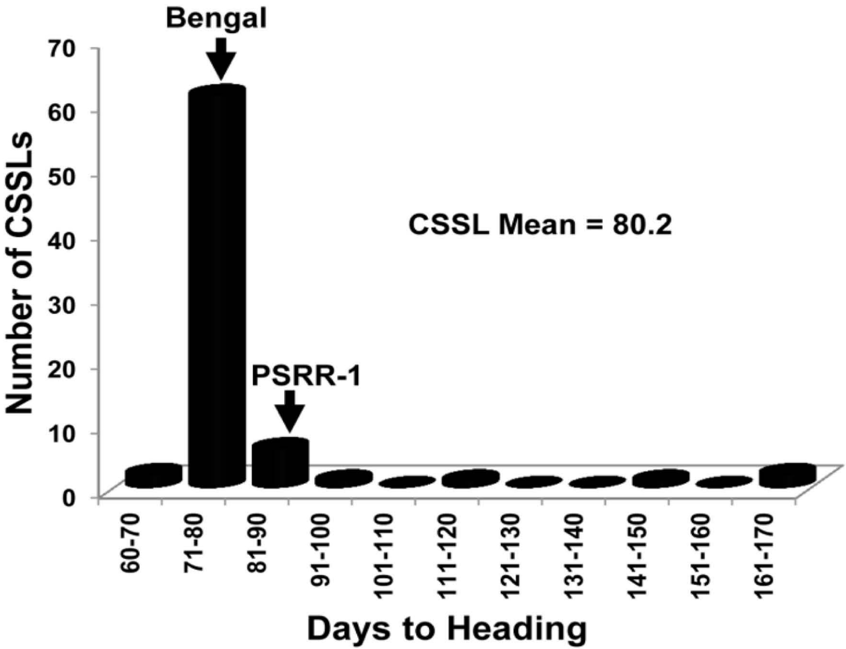

Supplementary Figure S4 Frequency distribution of days to heading in PSRR-1 IL population developed in the 'Bengal' background[35]. Phenotypic values of both parents are indicated by arrows.

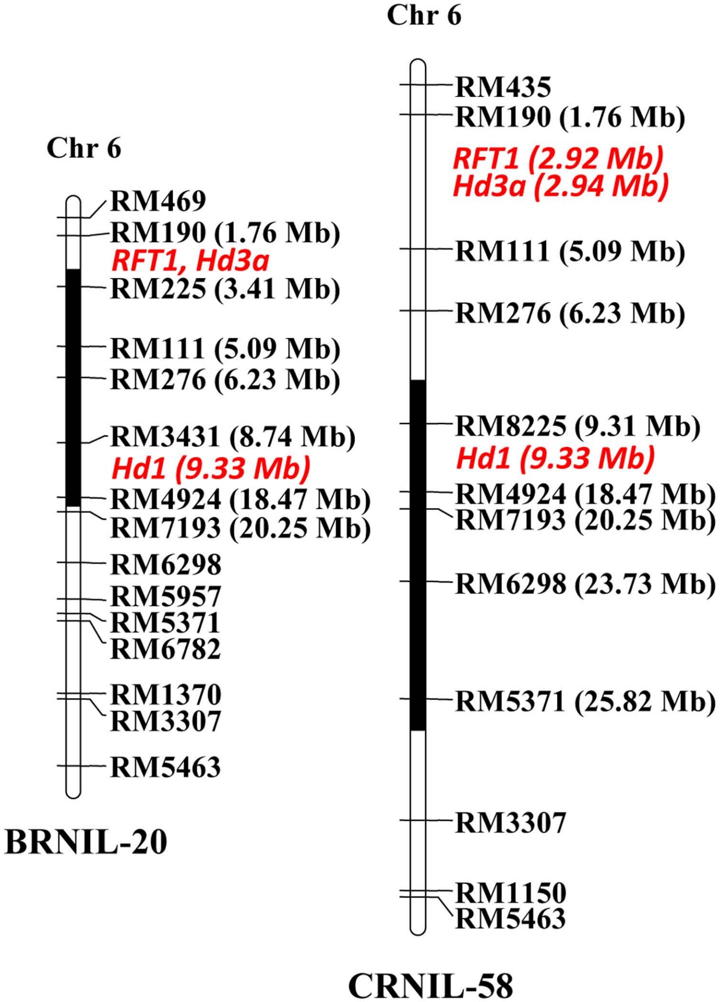

Supplementary Figure S5 Graphical genotyping of the photosensitive BRNIL-20 and CRNIL-58 with a single PSRR-1introgressed segment from chromosome 6. Chromosome maps were from the RIL linkage maps[34]. The peak position of QTL was at RM3431 (8.74 Mb) and RM8225 (9.31 Mb) in BR and CR-RIL populations, respectively. The physical map location of markers and flowering genes (Mb position based on reference genome) are given in parentheses.

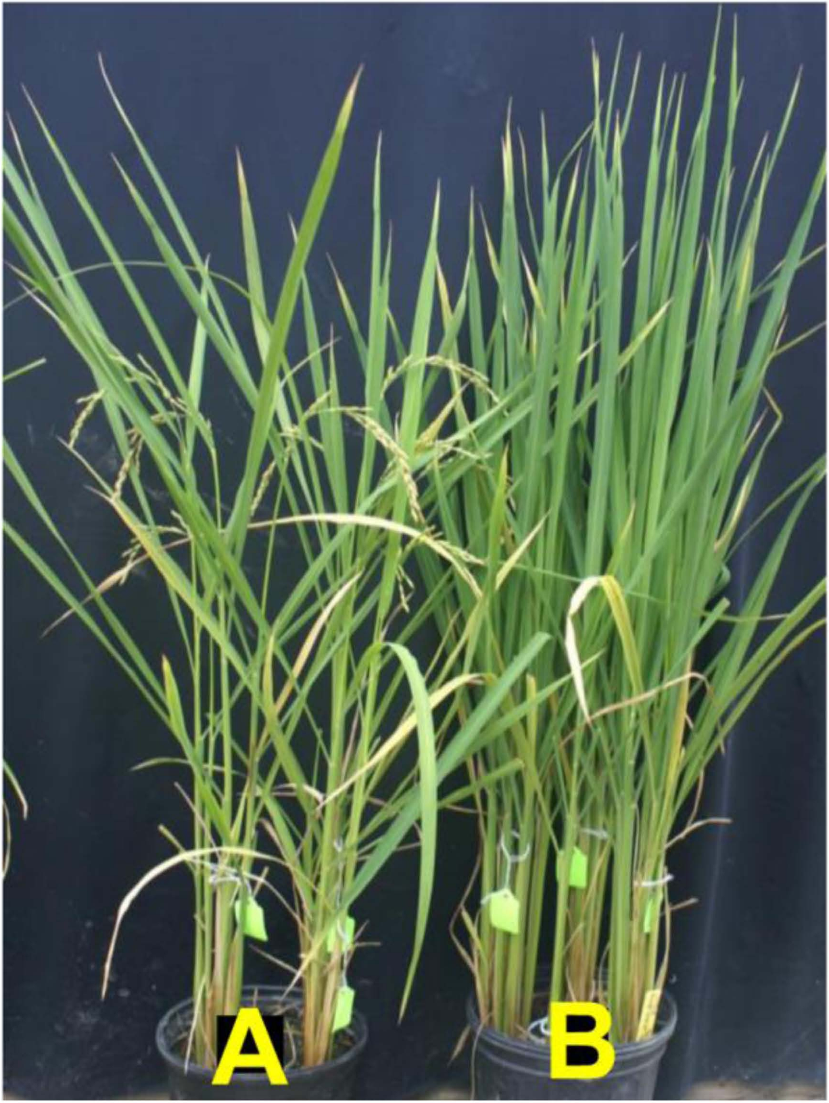

Supplementary Figure S6 Flowering in CRNIL-58 after exposure to 10 hr photoperiod (A) but no sign of flowering under natural long-day conditions (B). Planting date was April 18, 2012.

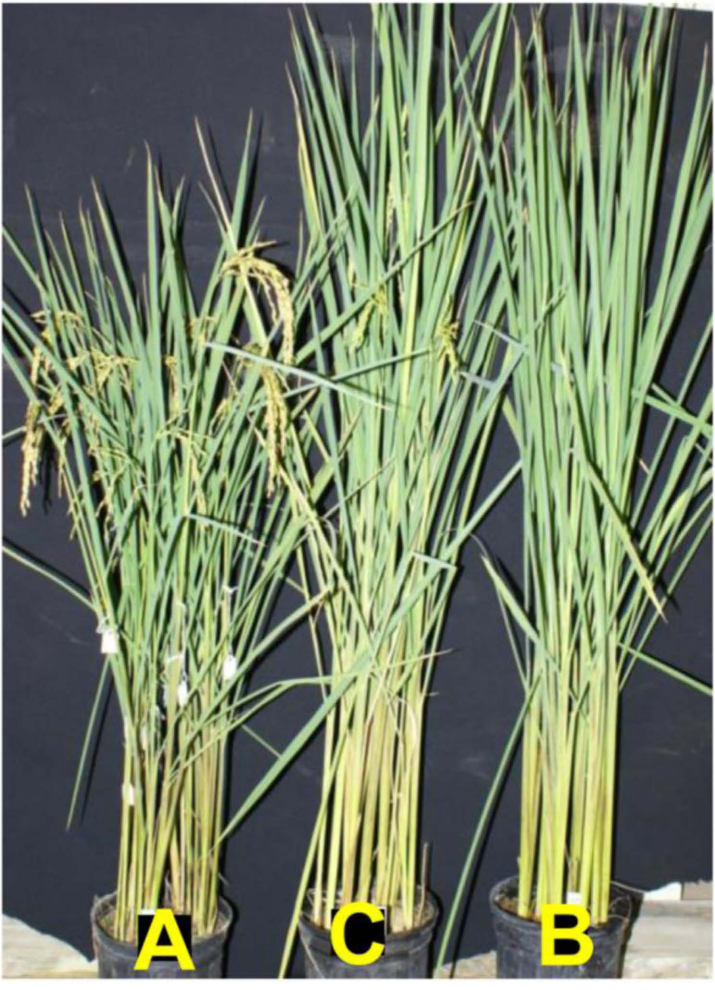

Supplementary Figure S7 Flowering response to natural long-day conditions in 'Bengal' (A), BRNIL-20 (B), and its F1 (C) 105 days after planting (planting date: April 18, 2012). The heading date of F1 plants was intermediate, whereas the NIL flowered late.

Supplementary Figure S8 Alignment of the Hd1 genomic (A), cDNA (B), predicted amino acid sequences (C), and domain analysis (D) of 'Nipponbare', 'Bengal', 'Cypress', and 'PSRR-1'.

(A) Hd1 Genomic alignment

```
                                  10        20        30        40        50        60        70        80
Hd1-Nipponbare gDNA.seq ATGAATTATAATTTTGGTGGCAACGTGTTCGACCAGGAGGTTGGAGTTGGAGGCGAAGGAGGAGGAGGAGGAGAGGGGAG 80
Hd1-Bengal gDNA.seq     ATGAATTATAATTTTGGTGGCAACGTGTTCGACCAGGAGGTTGGAGTTGGAGGCGAAGGAGGAGGAGGAGGAGAGGGGAG 80
Hd1-Cypress gDNA.seq    ATGAATTATAATTTTGGTGGCAACGTGTTCGACCAGGAGGTTGGAGTTGGAGGCGAAGGAGGAGGAGGAGGAGAGGGGAG 80
Hd1-PSRR gDNA.seq       ATGAATTATAATTTTGGTGGCAACGTGTTCGACCAGGAGGTTGGAGTTGGAGGCGAAGGAGGAGGAGGAGGAGAGGGGAG 80

                                  90       100       110       120       130       140       150       160
Hd1-Nipponbare gDNA.seq CGGCTGCCCATGGGCGCGGCCGTGCGACGGGTGCCGCGCGGCGCCGAGCGTGGTGTACTGCCGCGCGGACGCGGCGTACC 160
Hd1-Bengal gDNA.seq     CGGCTGCCCATGGGCGCGGCCGTGCGACGGGTGCCGCGCGGCGCCGAGCGTGGTGTACTGCCGCGCGGACGCGGCGTACC 160
Hd1-Cypress gDNA.seq    CGGCTGCCCATGGGCGCGGCCGTGCGACGGGTGCCGCGCGGCGCCGAGCGTGGTGTACTGCCGCGCGGACGCGGCGTACC 160
Hd1-PSRR gDNA.seq       CGGCTGCCCATGGGCGCGGCCGTGCGACGGGTGCCGCGCGGCGCCGAGCGTGGTGTACTGCCGCGCGGACGCGGCGTACC 160

                                 170       180       190       200       210       220       230       240
Hd1-Nipponbare gDNA.seq TGTGCGCGTCGTGCGACGCGCGGGTGCACGCGGCCAACCGCGTGGCGTCCCGCCACGAGCGCGTGCGGGTGTGCGAGGCC 240
Hd1-Bengal gDNA.seq     TGTGCGCGTCGTGCGACGCGCGGGTGCACGCGGCCAACTGCGTGGCGTCCCGCCACGAGCGCGTGCGGGTGTGCGAGGCC 240
Hd1-Cypress gDNA.seq    TGTGCGCGTCGTGCGACGCGCGGGTGCACGCGACCAACCGCGTGGCGTCCCGCCACGAGCGCGTGCGGGTGTGCGAGGCC 240
Hd1-PSRR gDNA.seq       TGTGCGCGTCGTGCGACGCGCGGGTGCACGCGGCCAACCGCGTGGCGTCCCGCCACGAGCGCGTGCGGGTGTGCGAGGCC 240

                                 250       260       270       280       290       300       310       320
Hd1-Nipponbare gDNA.seq TGCGAGCGCGCCCCGGCCGCGCTCGCGTGCCGCGCCGACGCCGCCGCGCTGTGCGTGGCGTGCGACGTGCAGGTGCACTC 320
Hd1-Bengal gDNA.seq     TGCGAGCAAGCCCCGGCCGCGCTCGCGTGCCGCGCCGACGCCGCCGCGCTGTGCGTGGCGTGCGACGTGCAGGTGCACTC 320
Hd1-Cypress gDNA.seq    TGCGAGCAAGCCCCGGCCGCGCTCGCGTGCCGCGCCGACGCCGCCGCGCTGTGCGTGGCGTGCGACGTGCAGGTGCACTC 320
Hd1-PSRR gDNA.seq       TGCGAGCGCGCCCCGGCCGCGCTCGCGTGCCGCGCCGACGCCGCCGCGCTGTGCGTGGCGTGCGACGTGCAGGTGCACTC 320

                                 330       340       350       360       370       380       390       400
Hd1-Nipponbare gDNA.seq CGCGAACCCGCTC------------------------------------CCGGCCATCACCATCCCGGCCACCTCCGTCC 364
Hd1-Bengal gDNA.seq     CGCGAACCCGCTCGCCAGGCGCCACCAGCGCGTCCCCGTCGCGCCGCTCCCGGCCATCACCATCCCGGCCACCTCCGTCC 400
Hd1-Cypress gDNA.seq    CGCGAACCCGCTCGCCAGGCGCCACCAGCGCGTCCCCGTCGCGCCGCTCCCGGCCATCACCATCCCGGCCACCTCCGTCC 400
Hd1-PSRR gDNA.seq       CGCGAACCCGCTCGCCAGGCGCCACCAGCGCGTCCCCGTCGCGCCGCTCCCGGCCATCACCATCCCGGCCACCTCCGTCC 400

                                 410       420       430       440       450       460       470       480
Hd1-Nipponbare gDNA.seq TCGCTGAGGCGGTGGTGGCCACCGCCACCGTCCTCGGCGACAAGGACGAGGAGGTGGACTCTTGGCTTCTCCTCTCCAAA 444
Hd1-Bengal gDNA.seq     TCGCTGAGGCGGTGGTGGCCACCGCCACCGTCCTCGGCGGCAAGGACGAGGAGGTGGACTCTTGGATTATCCTCTCCAAA 480
Hd1-Cypress gDNA.seq    TCGCTGAGGCGGTGGTGGCCACCGCCACCGTCCTCGGCGGCAAGGACGAGGAGGTGGACTCTTGGATTATCCTCTCCAAA 480
Hd1-PSRR gDNA.seq       TCGCTGAGGCGGTGGTGGCCACCGCCACCGTCCTCGGCGACAAGGACGAGGAGGTGGACTCTTGGCTTCTCCTCTCCAAA 480

                                 490       500       510       520       530       540       550       560
Hd1-Nipponbare gDNA.seq GATTCCGACAACAACAACAACAATAACAACA------------------------------------------------- 475
Hd1-Bengal gDNA.seq     GATTCCAACAACAACAACAACAATAACAACAGCAACAGCAGCAACAACGGCATGTATTTTGGTGAAGTCGATGAGTACTT 560
Hd1-Cypress gDNA.seq    GATTCCAACAACAACAACAACAATAACAACAGCAACAGCAGCAACAACGGCATGTATTTTGGTGAAGTCGATGAGTACTT 560
Hd1-PSRR gDNA.seq       GATTCCGACAACAACAACAACAATAACAACA------------------------------------------------- 511

                                 570       580       590       600       610       620       630       640
Hd1-Nipponbare gDNA.seq --------------------------------------------------------------------------ACAACG 481
Hd1-Bengal gDNA.seq     TGATCTTGTCAGGTACAATTCGTACTACGACAACAACAATAACGACAACAGCAACAGCAACAGCAGCAACAACGACAACG 640
Hd1-Cypress gDNA.seq    TGATCTTGTCAGGTACAATTCGTACTACGACAACAACAATAACGACAACAGCAACAGCAACAGCAGCAACAACGACAACG 640
Hd1-PSRR gDNA.seq       --------------------------------------------------------------------------ACAACG 517
```

```
                               650       660       670       680       690       700       710       720
Hdl-Nipponbare gDNA.seq  ACAACGACAATAACGACAACAACAACAGCAACAGCAGCAACAACGGCATGTATTTTGGTGAAGTCGATGAGTACTTTGAT  561
Hdl-Bengal gDNA.seq      ACAACGACAACGACAATAACAACAACAGCAACAGCAGCAACAACGGCATGTATTTTGGTGAAGTCGATGAGTACTTTGAT  720
Hdl-Cypress gDNA.seq     ACAACGACAACGACAATAACAACAACAGCAACAGCAGCAACAACGGCATGTATTTTGGTGAAGTCGATGAGTACTTTGAT  720
Hdl-PSRR gDNA.seq        ACAACGACAATAAGGACAACAACAACAGCAACAGCAGCAACAACGGCATGTATTTTGGTGAAGTCGATGAGTACTTTGAT  597

                               730       740       750       760       770       780       790       800
Hdl-Nipponbare gDNA.seq  CTTGTCGGGTACAATTCGTACTACGACAACCGCATCGAAAACAACCAAGATCGGCAGTATGGGATGCATGAACAGCAAGA  641
Hdl-Bengal gDNA.seq      CTTGTCGGGTACAATTCGTACTACGACAACCGCATCGAAAACAACCAAGATCAGCAGTATGGGATGCATGAACAGCAAGA  800
Hdl-Cypress gDNA.seq     CTTGTCGGGTACAATTCGTACTACGACAACCGCATCGAAAACAACCAAGATCAGCAGTATGGGATGCATGAACAGCAAGA  800
Hdl-PSRR gDNA.seq        CTTGTCGGGTACAATTCGTACTACGACAACCGCATCGAAAACAACCAAGATCAGCAGTATGGGATGCATGAACAGCAAGA  677

                               810       820       830       840       850       860       870       880
Hdl-Nipponbare gDNA.seq  GCAGCAGCAGCAGCAGCAGGAGATGCAAAAGGAGTTTGCAGAGAAGGAAGGGAGCGAGTGTGTGGTACCTTCACAGATCA  721
Hdl-Bengal gDNA.seq      GCAGCAGCAGCAGCAGCAGGAGATGCAAAAGGAGTTTGCAGAGAAGGAAGGGAGCGAGTGTGTGGTACCTTCACAGATCA  880
Hdl-Cypress gDNA.seq     GCAGCAGCAGCAGCAGCAGGAGATGCAAAAGGAGTTTGCAGAGAAGGAAGGGAGCGAGTGTGTGGTACCTTCACAGATCA  880
Hdl-PSRR gDNA.seq        GCAGCAGCAGCAGCAGCAGGAGATGCAAAAGGAGTTTGCAGAGAAGGAAGGGAGCGAGTGTGTGGTACCTTCACAGATCA  757

                               890       900       910       920       930       940       950       960
Hdl-Nipponbare gDNA.seq  CAATGCTGAGTGAGCAGCAGCATAGTGGTTATGGAGTTGTGGGAGCAGACCAGGCCGCCTCCATGACCGCCGGCGTCAGT  801
Hdl-Bengal gDNA.seq      CAATGCTGAGTGAGCAGCAGCATAGTGGTTATGGAGTTGTGGGAGCAGACCAGGCCGCCTCCATGACCGCCGGCGTCAGT  960
Hdl-Cypress gDNA.seq     CAATGCTGAGTGAGCAGCAGCATAGTGGTTATGGAGTTGTGGGAGCAGACCAGGCCGCCTCCATGACCGCCGGCGTCAGT  960
Hdl-PSRR gDNA.seq        CAATGCTGAGTGAGCAGCAGCATAGTGGTTATGGAGTTGTGGGAGCAGACCAGGCCGCCTCCATGACCGCCGGCGTCAGT  837

                               970       980       990      1000      1010      1020      1030      1040
Hdl-Nipponbare gDNA.seq  GCTTACACAGATTCCATCAGCAACAGCGTGAGTTCATCTATTACTAGCTGCAACTATTTTTTTTTCAGAGAATGAACATC  881
Hdl-Bengal gDNA.seq      GCTTACACAGATTCCATCAGCAACAGCGTGAGTTCATCTATTACTAGCTGCAACTATTTTTTTTTCAGAGAATGAACATC  1040
Hdl-Cypress gDNA.seq     GCTTACACAGATTCCATCAGCAACAGCGTGAGTTCATCTATTACTAGCTGCAACTATTTTTTTTTCAGAGAATGAACATC  1040
Hdl-PSRR gDNA.seq        GCTTACACAGATTCCATCAGCAACAGCGTGAGTTCATCTATTACTAGCTGCAACTATTTTTTTTTCAGAGAATGAACATC  917

                              1050      1060      1070      1080      1090      1100      1110      1120
Hdl-Nipponbare gDNA.seq  TATTACTGTTGTTAGTTAGTTGTTACTACATGCCACGTTGTCAATGTTTTAGAGTTCATACTAGTACTTTTGAGTGGAAA  961
Hdl-Bengal gDNA.seq      TATTACTGTTGTTAGTTAGTTGTTACTACATGCCACGTTGTCAATGTTTTAGAGTTCATACTAGTACTTTTTAGTGGAAA  1120
Hdl-Cypress gDNA.seq     TATTACTGTTGTTAGTTAGTTGTTACTACATGCCACGTTGTCAATGTTTTAGAGTTCATACTAGTACTTTTTAGTGGAAA  1120
Hdl-PSRR gDNA.seq        TATTACTGTTGTTAGTTAGTTGTTACTACATGCCACGTTGTCAATGTTTTAGAGTTCATACTAGTACTTTTGAGTGGAAA  997

                              1130      1140      1150      1160      1170      1180      1190      1200
Hdl-Nipponbare gDNA.seq  AACATTCTCCAAACAAAAGCTACTGTCTAACAAAATGAAGGGATAAATAAACAGATCTCAACAAGAAAACAAAGATACTT  1041
Hdl-Bengal gDNA.seq      AACATTCTCCAAACAAAAGCTACTGTCTAACAAAATGAAGGGATAAATAAACAGATCTCAACAAGAAAATAAAGATACTT  1200
Hdl-Cypress gDNA.seq     AACATTCTCCAAACAAAAGCTACTGTCTAACAAAATGAAGGGATAAATAAACAGATCTCAACAAGAAAATAAAGATACTT  1200
Hdl-PSRR gDNA.seq        AACATTCTCCAAACAAAAGCTACTGTCTAACAAAATGAAGGGATAAATAAACAGATCTCAACAAGAAAACAAAGATACTT  1077

                              1210      1220      1230      1240      1250      1260      1270      1280
Hdl-Nipponbare gDNA.seq  TTCTACTTCCAAGCTGCGATCTTTAGGCTGATTAAATGGAACCGATAAAAAAAATACTTTAAAGAAAAGTACACAATTGA  1121
Hdl-Bengal gDNA.seq      TTCTACTTCCAAGCTGCGATCTTTAGGCTGATTAAATGGAACCGATAAAAAAAATACTTTAAAGAAAAGTACACAATTGA  1280
Hdl-Cypress gDNA.seq     TTCTACTTCCAAGCTGCGATCTTTAGGCTGATTAAATGGAACCGATAAAAAAAATACTTTAAAGAAAAGTACACAATTGA  1280
Hdl-PSRR gDNA.seq        TTCTACTTCCAAGCTGCGATCTTTAGGCTGATTAAATGGAACCGATAAAAAAAATACTTTAAAGAAAAGTACACAATTGA  1157
```

```
                           1290      1300      1310      1320      1330      1340      1350      1360
Hdl-Nipponbare gDNA.seq   TCTTTAGGCAGACCAGTTGACTACTTCCTGTATTTCTAAGCATATACGATCCATGCTAACTCACTAATTGAAAAGAAGTG  1201
Hdl-Bengal gDNA.seq       TCTTTAGGCAGACCAGTTGACTAGTTCCTGTATTTCTAAGCATATACCATCCATGCTAACTCACTAATTGAAAAGAAGTG  1360
Hdl-Cypress gDNA.seq      TCTTTAGGCAGACCAGTTGACTAGTTCCTGTATTTCTAAGCATATACCATCCATGCTAACTCACTAATTGAAAAGAAGTG  1360
Hdl-PSRR gDNA.seq         TCTTTAGGCAGACCAGTTGACTACTTCCTGTATTTCTAAGCATATACGATCCATGCTAACTCACTAATTGAAAAGAAGTG  1237

                           1370      1380      1390      1400      1410      1420      1430      1440
Hdl-Nipponbare gDNA.seq   AGTTTGTTAACCTTTTATGTACACAGCAATCACCACACGAAAGACCTCATGAAAAGTAGGATAAGTGTAAGTGTAATTCA  1281
Hdl-Bengal gDNA.seq       AGTTTGTTAACCTTTTATGTACACAGCAATCACCACACGAAAGACCTCATGAAAAGTAGGATAAGTGTAAGTGTAATTCA  1440
Hdl-Cypress gDNA.seq      AGTTTGTTAACCTTTTATGTACACAGCAATCACCACACGAAAGACCTCATGAAAAGTAGGATAAGTGTAAGTGTAATTCA  1440
Hdl-PSRR gDNA.seq         AGTTTGTTAACCTTTTATGTACACAGCAATCACCACACGAAAGACCTCATGAAAAGTAGGATAAGTGTAAGTGTAATTCA  1317

                           1450      1460      1470      1480      1490      1500      1510      1520
Hdl-Nipponbare gDNA.seq   TATTTTATCCCAGTGCATAAATTTAAAATATCTTACTTTTGCGACAGTAAAAAAGATATTGGAAGTTTTTCTTATGTATG  1361
Hdl-Bengal gDNA.seq       TATTTTATCCCAGTGCATAAATTTAAAATATCTTACTTTTGCGACAGTAAAAAAGATATTGGAAGTTTTTCTTATGTATG  1520
Hdl-Cypress gDNA.seq      TATTTTATCCCAGTGCATAAATTTAAAATATCTTACTTTTGCGACAGTAAAAAAGATATTGGAAGTTTTTCTTATGTATG  1520
Hdl-PSRR gDNA.seq         TATTTTATCCCAGTGCATAAATTTAAAATATCTTACTTTTGCGACAGTAAAAAAGATATTGGAAGTTTTTCTTATGTATG  1397

                           1530      1540      1550      1560      1570      1580      1590      1600
Hdl-Nipponbare gDNA.seq   TAAAATTAAATTAAGCCCATCTATATATCATTGCAGGGTCTCTGACACCTGCAATCTCCTTATGATTCGCATATTTCAGT  1441
Hdl-Bengal gDNA.seq       TAAAATTAAATTAAGCCCATCTATATATCATTGCAGGGTCTCTGACACCTGCAATCTCCTTATGATTCGCATATTTCAGT  1600
Hdl-Cypress gDNA.seq      TAAAATTAAATTAAGCCCATCTATATATCATTGCAGGGTCTCTGACACCTGCAATCTCCTTATGATTCGCATATTTCAGT  1600
Hdl-PSRR gDNA.seq         TAAAATTAAATTAAGCCCATCTATATATCATTGCAGGGTCTCTGACACCTGCAATCTCCTTATGATTCGCATATTTCAGT  1477

                           1610      1620      1630      1640      1650      1660      1670      1680
Hdl-Nipponbare gDNA.seq   GACCATTTGCCGATTCCATCTCAGATATCTTTCTCATCAATGGAGGCGGGTATAGTACCAGACAGCACGGTGATAGATAT  1521
Hdl-Bengal gDNA.seq       GACCATTTGCCGATTCCATCTCAGATATCT--CTCATCAATGGAGGCGGGTATAGTACCAGACAGCACGGTGATAGATAT  1678
Hdl-Cypress gDNA.seq      GACCATTTGCCGATTCCATCTCAGATATCT--CTCATCAATGGAGGCGGGTATAGTACCAGACAGCACGGTGATAGATAT  1678
Hdl-PSRR gDNA.seq         GACCATTTGCCGATTCCATCTCAGATATCTTTCTCATCAATGGAGGCGGGTATAGTACCAGACAGCACGGTGATAGATAT  1557

                           1690      1700      1710      1720      1730      1740      1750      1760
Hdl-Nipponbare gDNA.seq   GCCAAATTCCAGAATCCTGACACCTGCTGGAGCAATCAATCTCTTCTCAGGTCCCTCGCTTCAGATGTCCCTTCACTTCA  1601
Hdl-Bengal gDNA.seq       GCCAAATTCCAGCATCCTGACACCTGCTGGAGCAATCAATCTCTTCTCAGGTCCCTCGCTTCAGATGTCCCTTCACTTCA  1758
Hdl-Cypress gDNA.seq      GCCAAATTCCAGCATCCTGACACCTGCTGGAGCAATCAATCTCTTCTCAGGTCCCTCGCTTCAGATGTCCCTTCACTTCA  1758
Hdl-PSRR gDNA.seq         GCCAAATTCCAGAATCCTGACACCTGCTGGAGCAATCAATCTCTTCTCAGGTCCCTCGCTTCAGATGTCCCTTCACTTCA  1637

                           1770      1780      1790      1800      1810      1820      1830      1840
Hdl-Nipponbare gDNA.seq   GCTCCATGGACAGGGAGGCCAGGGTGCTCAGGTACAGGGAGAAGAAGAAGGCCAGGAAGTTTGAGAAGACAATACGTTAT  1681
Hdl-Bengal gDNA.seq       GCTCCATGGACAGGGAGGCCAGGGTGCTCAGGTACAGGGAGAAGAAGAAGGCCAGGAAGTTTGAGAAGACAATACGTTAT  1838
Hdl-Cypress gDNA.seq      GCTCCATGGACAGGGAGGCCAGGGTGCTCAGGTACAGGGAGAAGAAGAAGGCCAGGAAGTTTGAGAAGACAATACGTTAT  1838
Hdl-PSRR gDNA.seq         GCTCCATGGACAGGGAGGCCAGGGTGCTCAGGTACAGGGAGAAGAAGAAGGCCAGGAAGTTTGAGAAGACAATACGTTAT  1717

                           1850      1860      1870      1880      1890      1900      1910      1920
Hdl-Nipponbare gDNA.seq   GAAACAAGAAAGGCGTATGCAGAGGCACGACCCCGGATCAAGGGCCGTTTCGCCAAGAGATCAGATGTGCAGATCGAAGT  1761
Hdl-Bengal gDNA.seq       GAAACAAGAAAGGCGTATGCAGAGGCACGACCCCGGATCAAGGGCCGTTTCGCCAAGAGATCAGATGTGCAGATCGAAGT  1918
Hdl-Cypress gDNA.seq      GAAACAAGAAAGGCGTATGCAGAGGCACGACCCCGGATCAAGGGCCGTTTCGCCAAGAGATCAGATGTGCAGATCGAAGT  1918
Hdl-PSRR gDNA.seq         GAAACAAGAAAGGCGTATGCAGAGGCACGACCCCGGATCAAGGGCCGTTTCGCCAAGAGATCAGATGTGCAGATCGAAGT  1797

                           1930      1940      1950      1960      1970      1980
Hdl-Nipponbare gDNA.seq   GGACCAGATGTTCTCCACTGCAGCTCTATCTGACGGTAGCTATGGTACTGTTCCATGGTTCTGA                  1825
Hdl-Bengal gDNA.seq       GGACCAGATGTTCTCCACTGCAGCTCTATCTGACAGTAGCTATGGTACTGTTCCATGGTTCTGA                  1982
Hdl-Cypress gDNA.seq      GGACCAGATGTTCTCCACTGCAGCTCTATCTGACAGTAGCTATGGTACTGTTCCATGGTTCTGA                  1982
Hdl-PSRR gDNA.seq         GGACCAGATGTTCTCCACTGCAGCTCTATCTGACGGTAGCTATGGTACTGTTCCATGGTTCTGA                  1861
```

(B) Hd1 CDS alignment

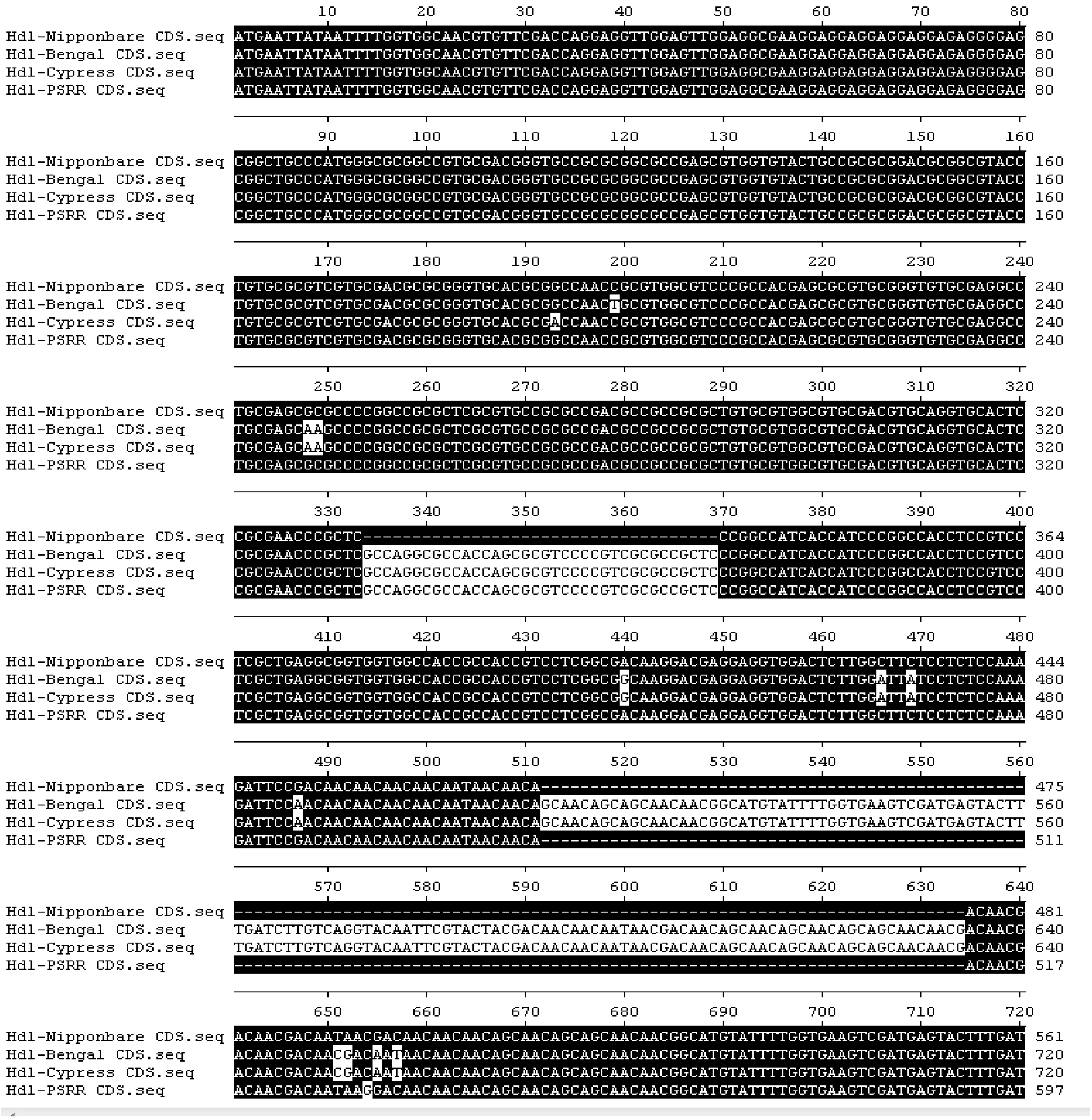

```
                          730       740       750       760       770       780       790       800
Hdl-Nipponbare CDS.seq CTTGTCGGGTACAATTCGTACTACGACAACCGCATCGAAAACAACCAAGATCGGCAGTATGGGATGCATGAACAGCAAGA 641
Hdl-Bengal CDS.seq     CTTGTCGGGTACAATTCGTACTACGACAACCGCATCGAAAACAACCAAGATCAGCAGTATGGGATGCATGAACAGCAAGA 800
Hdl-Cypress CDS.seq    CTTGTCGGGTACAATTCGTACTACGACAACCGCATCGAAAACAACCAAGATCAGCAGTATGGGATGCATGAACAGCAAGA 800
Hdl-PSRR CDS.seq       CTTGTCGGGTACAATTCGTACTACGACAACCGCATCGAAAACAACCAAGATCAGCAGTATGGGATGCATGAACAGCAAGA 677

                          810       820       830       840       850       860       870       880
Hdl-Nipponbare CDS.seq GCAGCAGCAGCAGCAGCAGGAGATGCAAAAGGAGTTTGCAGAGAAGGAAGGGAGCGAGTGTGTGGTACCTTCACAGATCA 721
Hdl-Bengal CDS.seq     GCAGCAGCAGCAGCAGCAGGAGATGCAAAAGGAGTTTGCAGAGAAGGAAGGGAGCGAGTGTGTGGTACCTTCACAGATCA 880
Hdl-Cypress CDS.seq    GCAGCAGCAGCAGCAGCAGGAGATGCAAAAGGAGTTTGCAGAGAAGGAAGGGAGCGAGTGTGTGGTACCTTCACAGATCA 880
Hdl-PSRR CDS.seq       GCAGCAGCAGCAGCAGCAGGAGATGCAAAAGGAGTTTGCAGAGAAGGAAGGGAGCGAGTGTGTGGTACCTTCACAGATCA 757

                          890       900       910       920       930       940       950       960
Hdl-Nipponbare CDS.seq CAATGCTGAGTGAGCAGCAGCATAGTGGTTATGGAGTTGTGGGAGCAGACCAGGCCGCCTCCATGACCGCCGGCGTCAGT 801
Hdl-Bengal CDS.seq     CAATGCTGAGTGAGCAGCAGCATAGTGGTTATGGAGTTGTGGGAGCAGACCAGGCCGCCTCCATGACCGCCGGCGTCAGT 960
Hdl-Cypress CDS.seq    CAATGCTGAGTGAGCAGCAGCATAGTGGTTATGGAGTTGTGGGAGCAGACCAGGCCGCCTCCATGACCGCCGGCGTCAGT 960
Hdl-PSRR CDS.seq       CAATGCTGAGTGAGCAGCAGCATAGTGGTTATGGAGTTGTGGGAGCAGACCAGGCCGCCTCCATGACCGCCGGCGTCAGT 837

                          970       980       990      1000      1010      1020      1030      1040
Hdl-Nipponbare CDS.seq GCTTACACAGATTCCATCAGCAACAGCATATCTTTCTCATCAATGGAGGCGGGTATAGTACCAGACAGCACGGTGATAGA 881
Hdl-Bengal CDS.seq     GCTTACACAGATTCCATCAGCAACAGCATATC--TCTCATCAATGGAGGCGGGTATAGTACCAGACAGCACGGTGATAGA 1038
Hdl-Cypress CDS.seq    GCTTACACAGATTCCATCAGCAACAGCATATC--TCTCATCAATGGAGGCGGGTATAGTACCAGACAGCACGGTGATAGA 1038
Hdl-PSRR CDS.seq       GCTTACACAGATTCCATCAGCAACAGCATATCTTTCTCATCAATGGAGGCGGGTATAGTACCAGACAGCACGGTGATAGA 917

                         1050      1060      1070      1080      1090      1100      1110      1120
Hdl-Nipponbare CDS.seq TATGCCAAATTCCAGAATCCTGACACCTGCTGGAGCAATCAATCTCTTCTCAGGTCCCTCGCTTCAGATGTCCCTTCACT 961
Hdl-Bengal CDS.seq     TATGCCAAATTCCAGCATCCTGACACCTGCTGGAGCAATCAATCTCTTCTCAGGTCCCTCGCTTCAGATGTCCCTTCACT 1118
Hdl-Cypress CDS.seq    TATGCCAAATTCCAGCATCCTGACACCTGCTGGAGCAATCAATCTCTTCTCAGGTCCCTCGCTTCAGATGTCCCTTCACT 1118
Hdl-PSRR CDS.seq       TATGCCAAATTCCAGAATCCTGACACCTGCTGGAGCAATCAATCTCTTCTCAGGTCCCTCGCTTCAGATGTCCCTTCACT 997

                         1130      1140      1150      1160      1170      1180      1190      1200
Hdl-Nipponbare CDS.seq TCAGCTCCATGGACAGGGAGGCCAGGGTGCTCAGGTACAGGGAGAAGAAGAAGGCCAGGAAGTTTGAGAAGACAATACGT 1041
Hdl-Bengal CDS.seq     TCAGCTCCATGGACAGGGAGGCCAGGGTGCTCAGGTACAGGGAGAAGAAGAAGGCCAGGAAGTTTGA              1185
Hdl-Cypress CDS.seq    TCAGCTCCATGGACAGGGAGGCCAGGGTGCTCAGGTACAGGGAGAAGAAGAAGGCCAGGAAGTTTGA              1185
Hdl-PSRR CDS.seq       TCAGCTCCATGGACAGGGAGGCCAGGGTGCTCAGGTACAGGGAGAAGAAGAAGGCCAGGAAGTTTGAGAAGACAATACGT 1077

                         1210      1220      1230      1240      1250      1260      1270      1280
Hdl-Nipponbare CDS.seq TATGAAACAAGAAAGGCGTATGCAGAGGCACGACCCCGGATCAAGGGCCGTTTCGCCAAGAGATCAGATGTGCAGATCGA 1121
Hdl-Bengal CDS.seq                                                                                     1185
Hdl-Cypress CDS.seq                                                                                    1185
Hdl-PSRR CDS.seq       TATGAAACAAGAAAGGCGTATGCAGAGGCACGACCCCGGATCAAGGGCCGTTTCGCCAAGAGATCAGATGTGCAGATCGA 1157

                         1290      1300      1310      1320      1330      1340
Hdl-Nipponbare CDS.seq AGTGGACCAGATGTTCTCCACTGCAGCTCTATCTGACGGTAGCTATGGTACTGTTCCATGGTTCTGA              1188
Hdl-Bengal CDS.seq                                                                                     1185
Hdl-Cypress CDS.seq                                                                                    1185
Hdl-PSRR CDS.seq       AGTGGACCAGATGTTCTCCACTGCAGCTCTATCTGACGGTAGCTATGGTACTGTTCCATGGTTCTGA              1224
```

(C) Hd1 Protein alignment

```
                                          10         20         30         40         50         60         70         80
Hd1-Nipponbare Protein Seq.pro  MNYNFGGNVFDQEVGVGGEGGGGGEGSGCPWARPCDGCRAAPSVVYCRADAAYLCASCDARVHAANRVASRHERVRVCEA  80
Hd1-Bengal Protein Seq.pro      MNYNFGGNVFDQEVGVGGEGGGGGEGSGCPWARPCDGCRAAPSVVYCRADAAYLCASCDARVHAANCVASRHERVRVCEA  80
Hd1-Cypress Protein Seq.pro     MNYNFGGNVFDQEVGVGGEGGGGGEGSGCPWARPCDGCRAAPSVVYCRADAAYLCASCDARVHATNRVASRHERVRVCEA  80
Hd1-PSRR Protein Seq.pro        MNYNFGGNVFDQEVGVGGEGGGGGEGSGCPWARPCDGCRAAPSVVYCRADAAYLCASCDARVHAANRVASRHERVRVCEA  80

                                          90        100        110        120        130        140        150        160
Hd1-Nipponbare Protein Seq.pro  CERAPAALACRADAAALCVACDVQVHSANPL------------PAITIPATSVLAEAVVATATVLGDKDEEVDSWLLLSK  148
Hd1-Bengal Protein Seq.pro      CEQAPAALACRADAAALCVACDVQVHSANPLARRHQRVPVAPLPAITIPATSVLAEAVVATATVLGGKDEEVDSWIILSK  160
Hd1-Cypress Protein Seq.pro     CEQAPAALACRADAAALCVACDVQVHSANPLARRHQRVPVAPLPAITIPATSVLAEAVVATATVLGGKDEEVDSWIILSK  160
Hd1-PSRR Protein Seq.pro        CERAPAALACRADAAALCVACDVQVHSANPLARRHQRVPVAPLPAITIPATSVLAEAVVATATVLGDKDEEVDSWLLLSK  160

                                         170        180        190        200        210        220        230        240
Hd1-Nipponbare Protein Seq.pro  DSDNNNNNNN-----------------------------------------NNDNDNNDNNNSNSSNNGMYFGEVDEYFD  187
Hd1-Bengal Protein Seq.pro      DSNNNNNNNNSNSSNNGMYFGEVDEYFDLVRYNSYYDNNNNDNSNSNSSNNDNDNDNDNNNNSNSSNNGMYFGEVDEYFD  240
Hd1-Cypress Protein Seq.pro     DSNNNNNNNNSNSSNNGMYFGEVDEYFDLVRYNSYYDNNNNDNSNSNSSNNDNDNDNDNNNNSNSSNNGMYFGEVDEYFD  240
Hd1-PSRR Protein Seq.pro        DSDNNNNNNN-----------------------------------------NNDNDNKDNNNSNSSNNGMYFGEVDEYFD  199

                                         250        260        270        280        290        300        310        320
Hd1-Nipponbare Protein Seq.pro  LVGYNSYYDNRIENNQDRQYGMHEQQEQQQQQQEMQKEFAEKEGSECVVPSQITMLSEQQHSGYGVVGADQAASMTAGVS  267
Hd1-Bengal Protein Seq.pro      LVGYNSYYDNRIENNQDQQYGMHEQQEQQQQQQEMQKEFAEKEGSECVVPSQITMLSEQQHSGYGVVGADQAASMTAGVS  320
Hd1-Cypress Protein Seq.pro     LVGYNSYYDNRIENNQDQQYGMHEQQEQQQQQQEMQKEFAEKEGSECVVPSQITMLSEQQHSGYGVVGADQAASMTAGVS  320
Hd1-PSRR Protein Seq.pro        LVGYNSYYDNRIENNQDQQYGMHEQQEQQQQQQEMQKEFAEKEGSECVVPSQITMLSEQQHSGYGVVGADQAASMTAGVS  279

                                         330        340        350        360        370        380        390        400
Hd1-Nipponbare Protein Seq.pro  AYTDSISNSISFSSMEAGIVPDSTVIDMPNSRILTPAGAINLFSGPSLQMSLHFSSMDREARVLRYREKKKARKFEKTIR  347
Hd1-Bengal Protein Seq.pro      AYTDSISNSIS------------------------------LINGGG------YSTRQHGDRYAKFQHPDTCWSNQSLLR  364
Hd1-Cypress Protein Seq.pro     AYTDSISNSIS------------------------------LINGGG------YSTRQHGDRYAKFQHPDTCWSNQSLLR  364
Hd1-PSRR Protein Seq.pro        AYTDSISNSISFSSMEAGIVPDSTVIDMPNSRILTPAGAINLFSGPSLQMSLHFSSMDREARVLRYREKKKARKFEKTIR  359

                                         410        420        430        440
Hd1-Nipponbare Protein Seq.pro  YETRKAYAEARPRIKGRFAKRSDVQIEVDQMFSTAALSDGSYGTVPWF.                                396
Hd1-Bengal Protein Seq.pro      -----SLASDVPSLQLHGQGGQGAQVQGEEEGQEV.                                             395
Hd1-Cypress Protein Seq.pro     -----SLASDVPSLQLHGQGGQGAQVQGEEEGQEV.                                             395
Hd1-PSRR Protein Seq.pro        YETRKAYAEARPRIKGRFAKRSDVQIEVDQMFSTAALSDGSYGTVPWF.                                408
```

Decoration 'Decoration #1': Shade (with solid black) residues that match the Consensus exactly.

(D) Domain analysis in Hd1 (Conserved domain search in NCBI website)

Nipponbare Hd1

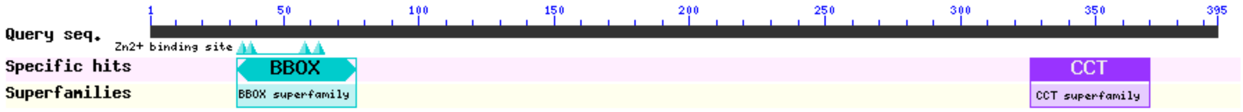

Name	Accession	Description	Interval	E-value
BBOX	cd00021	B-Box-type zinc finger; zinc binding domain (CHC3H2); often present in combination with other ...	33-77	2.19e-09

B-Box-type zinc finger; zinc binding domain (CHC3H2); often present in combination with other motifs, like RING zinc finger, NHL motif, coiled-coil or RFP domain in functionally unrelated proteins, most likely mediating protein-protein interaction.

:**Pssm-ID:** 237988 **Cd Length:** 39 **Bit Score:** 51.13 **E-value:** 2.19e-09

```
                                            10        20        30        40
                                    ....*....|....*....|....*....|....*....|....*
02c7b4269b65075ac52abbf17461a   33  RPCDGCRAAPSVVYCRADAAYLCASCDARVHaanrvaSRHERVRV  77
                                 1  RLCDEHGEEPLSLFCETDRALLCVDCDLSVH------SGHRRVPL  39
```

Name	Accession	Description	Interval	E-value
CCT	pfam06203	CCT motif; This short motif is found in a number of plant proteins. It is rich in basic amino ...	326-370	3.61e-24

CCT motif; This short motif is found in a number of plant proteins. It is rich in basic amino acids and has been called a CCT motif after Co, Col and Toc1. The CCT motif is about 45 amino acids long and contains a putative nuclear localisation signal within the second half of the CCT motif. Toc1 mutants have been identified in this region.

:**Pssm-ID:** 203407 **Cd Length:** 45 **Bit Score:** 92.67 **E-value:** 3.61e-24

```
                                            10        20        30        40
                                    ....*....|....*....|....*....|....*....|....*
02c7b4269b65075ac52abbf17461a  326  REARVLRYREKKKARKFEKTIRYETRKAYAEARPRIKGRFAKRSD  370
                                 1  REAALLRYKEKRKTRKFDKKIRYASRKAVAESRPRVKGRFVKQSE  45
```

Bengal Hd1

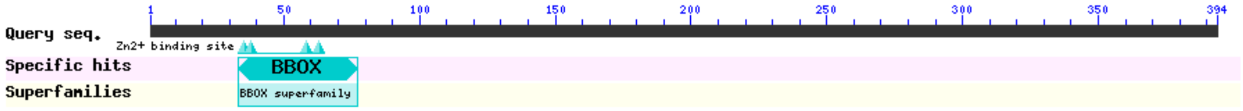

Name	Accession	Description	Interval	E-value
BBOX	cd00021	B-Box-type zinc finger; zinc binding domain (CHC3H2); often present in combination with other ...	33-77	2.08e-08

B-Box-type zinc finger; zinc binding domain (CHC3H2); often present in combination with other motifs, like RING zinc finger, NHL motif, coiled-coil or RFP domain in functionally unrelated proteins, most likely mediating protein-protein interaction.

:**Pssm-ID:** 237988 **Cd Length:** 39 **Bit Score:** 48.44 **E-value:** 2.08e-08

```
                                            10        20        30        40
                                    ....*....|....*....|....*....|....*....|....*
af0c4dfdf37fcca6bb11d7740804a   33  RPCDGCRAAPSVVYCRADAAYLCASCDARVHaancvaSRHERVRV  77
                                 1  RLCDEHGEEPLSLFCETDRALLCVDCDLSVH------SGHRRVPL  39
```

Cypress Hd1

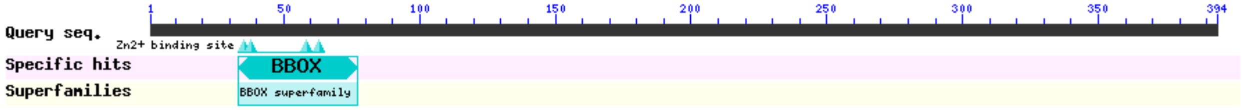

Name	Accession	Description	Interval	E-value
BBOX	cd00021	B-Box-type zinc finger; zinc binding domain (CHC3H2); often present in combination with other ...	33-77	1.25e-08

B-Box-type zinc finger; zinc binding domain (CHC3H2); often present in combination with other motifs, like RING zinc finger, NHL motif, coiled-coil or RFP domain in functionally unrelated proteins, most likely mediating protein-protein interaction.

:**Pssm-ID:** 237988 **Cd Length:** 39 **Bit Score:** 49.21 **E-value:** 1.25e-08

```
                                            10        20        30        40
                                     ....*....|....*....|....*....|....*....|....*
1dd7cc79789718d4c6f329cc7a6f3    33  RPCDGCRAAPSVVYCRADAAYLCASCDARVHatnrvaSRHERVRV  77
                                  1  RLCDEHGEEPLSLFCETDRALLCVDCDLSVH------SGHRRVPL  39
```

PSRR-1 Hd1

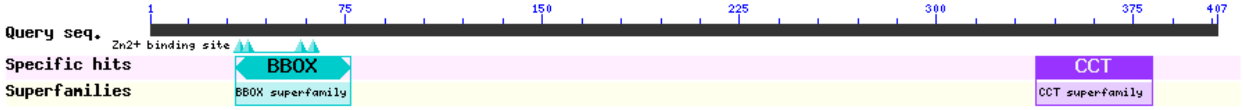

Name	Accession	Description	Interval	E-value
BBOX	cd00021	B-Box-type zinc finger; zinc binding domain (CHC3H2); often present in combination with other ...	33-77	3.12e-09

B-Box-type zinc finger; zinc binding domain (CHC3H2); often present in combination with other motifs, like RING zinc finger, NHL motif, coiled-coil or RFP domain in functionally unrelated proteins, most likely mediating protein-protein interaction.
:**Pssm-ID:** 237988 **Cd Length:** 39 **Bit Score:** 50.75 **E-value:** 3.12e-09

```
                                            10        20        30        40
                                     ....*....|....*....|....*....|....*....|....*
c93ef2a3d20eae089bfb1688ff51c    33  RPCDGCRAAPSVVYCRADAAYLCASCDARVHaanrvaSRHERVRV  77
                                  1  RLCDEHGEEPLSLFCETDRALLCVDCDLSVH------SGHRRVPL  39
```

Name	Accession	Description	Interval	E-value
CCT	pfam06203	CCT motif; This short motif is found in a number of plant proteins. It is rich in basic amino ...	338-382	5.08e-24

CCT motif; This short motif is found in a number of plant proteins. It is rich in basic amino acids and has been called a CCT motif after Co, Col and Toc1. The CCT motif is about 45 amino acids long and contains a putative nuclear localisation signal within the second half of the CCT motif. Toc1 mutants have been identified in this region.

:**Pssm-ID:** 203407 **Cd Length:** 45 **Bit Score:** 92.28 **E-value:** 5.08e-24

```
                                            10        20        30        40
                                     ....*....|....*....|....*....|....*....|....*
c93ef2a3d20eae089bfb1688ff51c   338  REARVLRYREKKKARKFEKTIRYETRKAYAEARPRIKGRFAKRSD  382
                                  1  REAALLRYKEKRKTRKFDKKIRYASRKAVAESRPRVKGRFVKQSE  45
```

Presence of CCT motif and B-BOX type zinc finger domains in Nipponbare, PSRR-1, Cypress, and Bengal

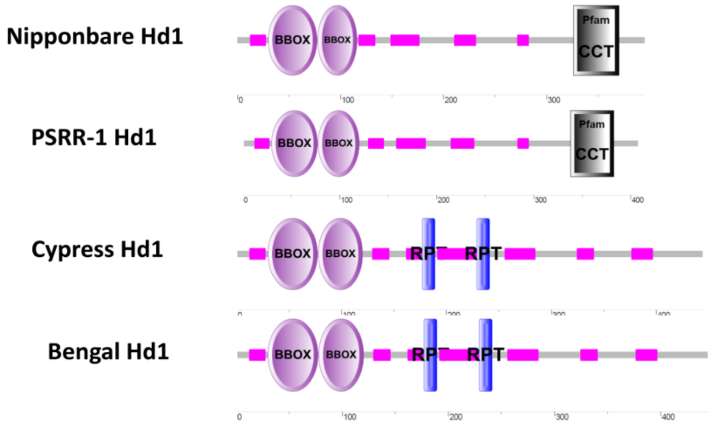

Hd3a promoter (1823 bp) and 5' UTR (152 bp) region

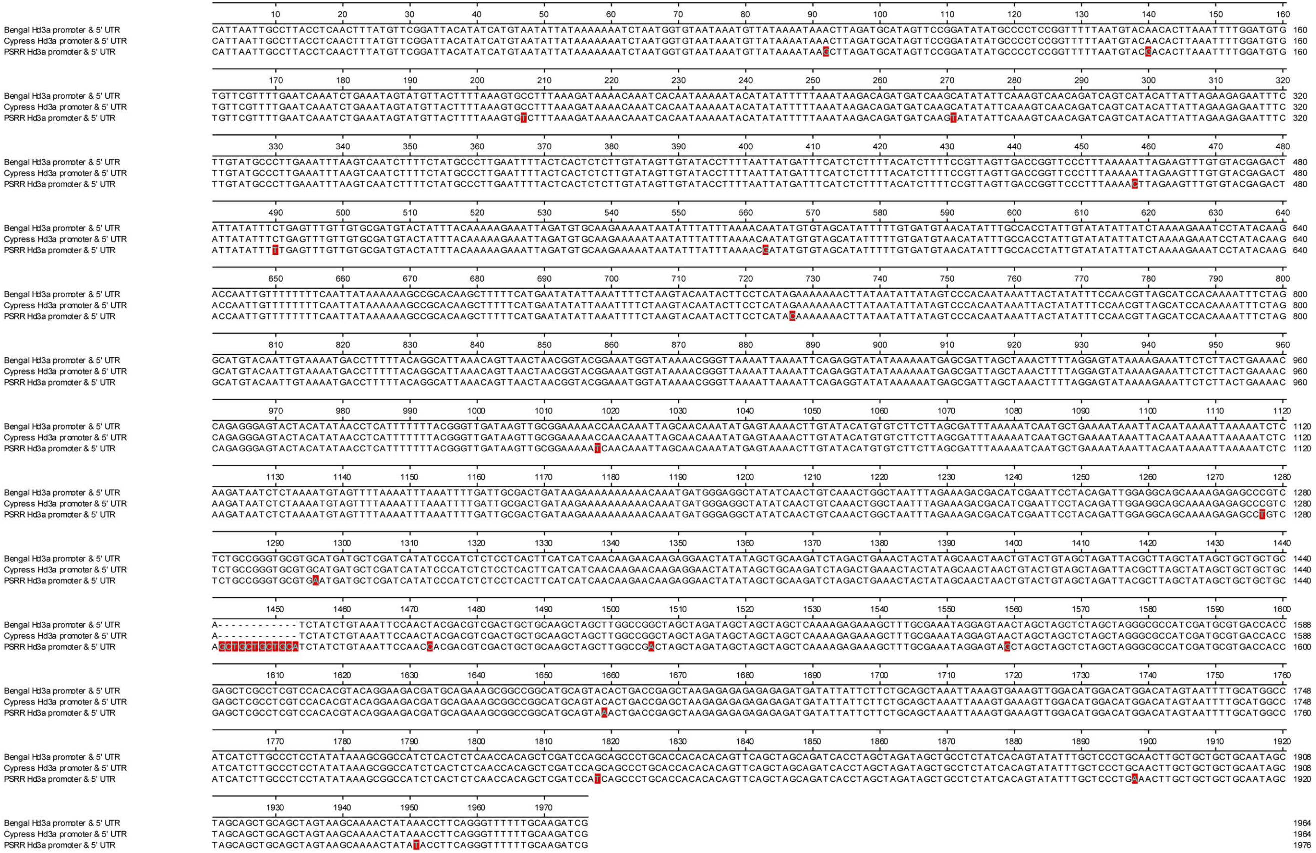

Supplementary Figure S9 Alignment of Hd3a promoter and 5' UTR regions of 'Bengal', 'Cypress', and 'PSRR-1'.

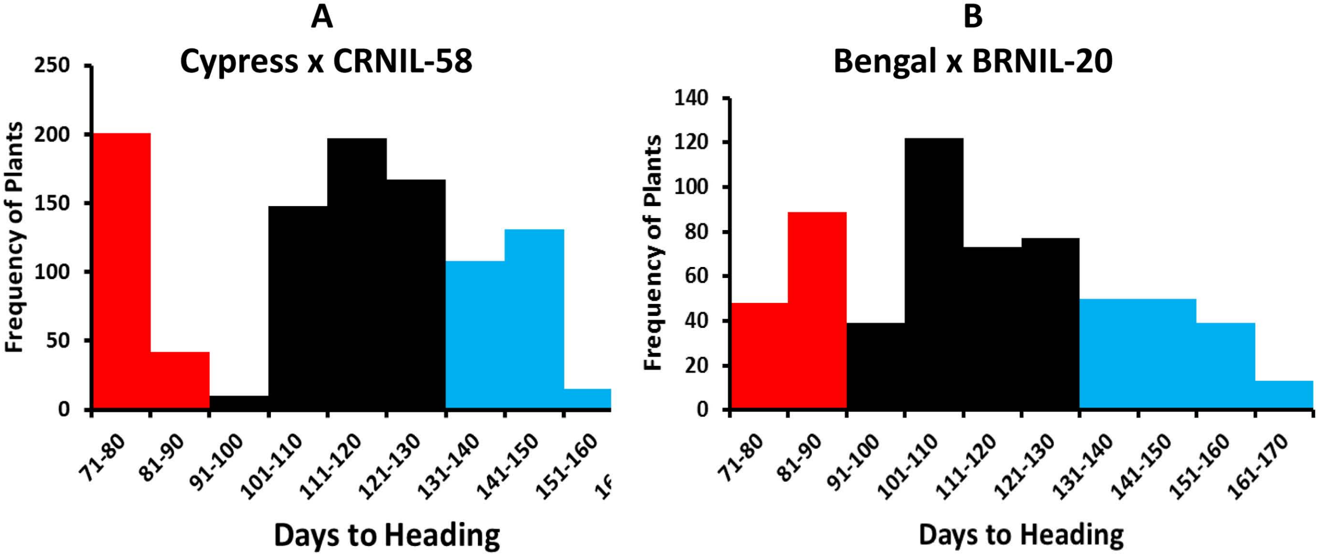

Supplementary Figure S10 Frequency distribution of plants in F_2 populations from the crosses, Cypress x CRNIL-58 (n=1020) (A) and Bengal x BRNIL-20 (n=600) (B) for heading date in a field experiment in 2014. The F_2 plants flowering in ≤90, 91-130, and >130 days were classified as early (E), intermediate (I), and late (L), respectively. The segregation ratio fit into 1:2:1 (Chisquare values were 0.564 and 1.153 for the Cypress x CRNIL-58 and Bengal x BRNIL-20 F_2 populations, respectively.

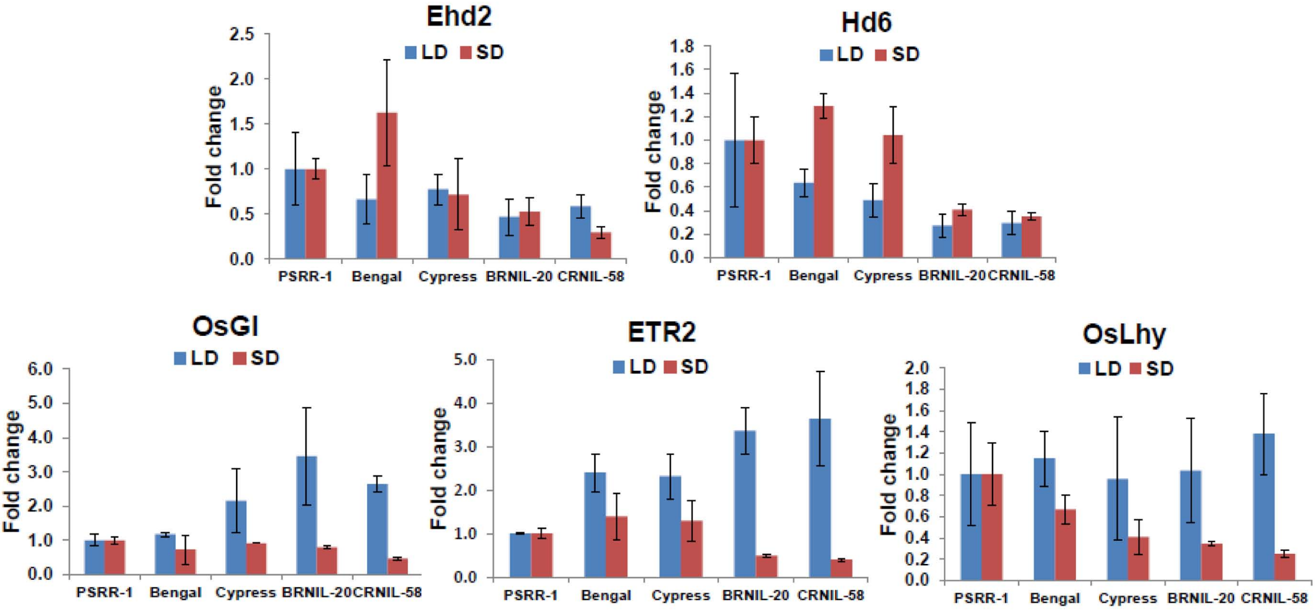

Supplementary Figure S11 Quantitative RT-PCR analysis of flowering pathway genes (*Ehd2, Hd6, OsGI, ETR2,* and *OsLhy*) in parents and NILs under natural LD and SD conditions. Transcript levels in leaves sampled 55 d after planting were measured in three biological replicates with three technical replications. The mean values of the relative expression levels of genes in BRNIL-20, CRNIL-58, 'Bengal', and 'Cypress' were compared with 'PSRR-1' and standard errors were indicated by the error bars. The rice *Actin1* gene was used as the internal control for normalization.

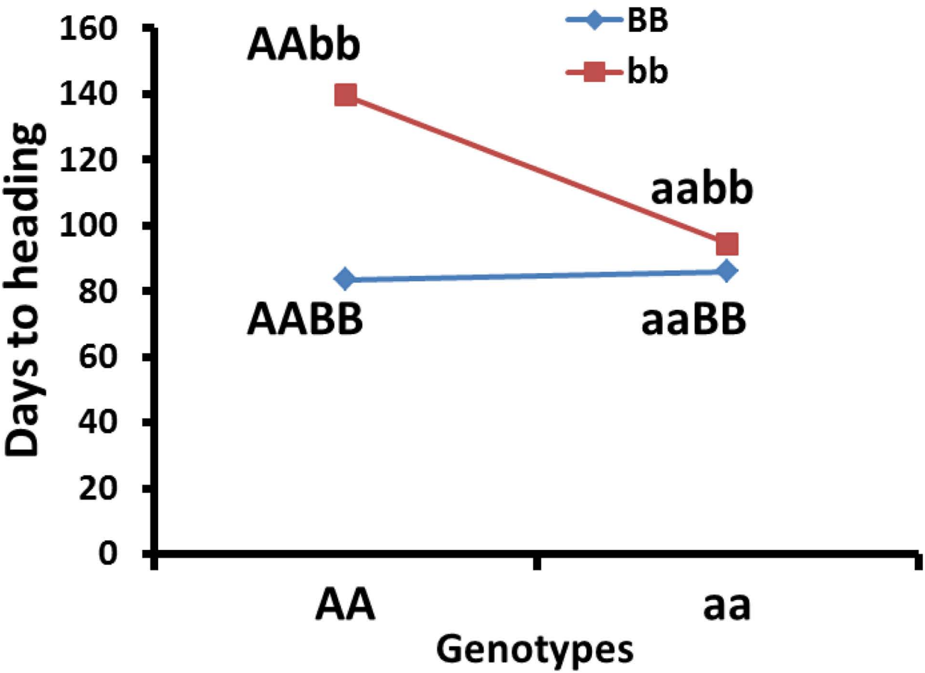

Supplementary Figure S12 Validation of the two locus interaction for photoperiodic flowering in the BR-RIL population. The QTLs *qHD6*^{BR} and *qHD7-1*^{BR} were represented by their nearest markers RM3431 and RM214, respectively. The labels AA and aa on horizontal axis represented 'PSRR' homozygotes and 'Bengal' homozygotes, respectively, for marker RM3431. BB and bb represent 'PSRR' homozygotes and 'Bengal' homozygotes, respectively, for marker RM214.

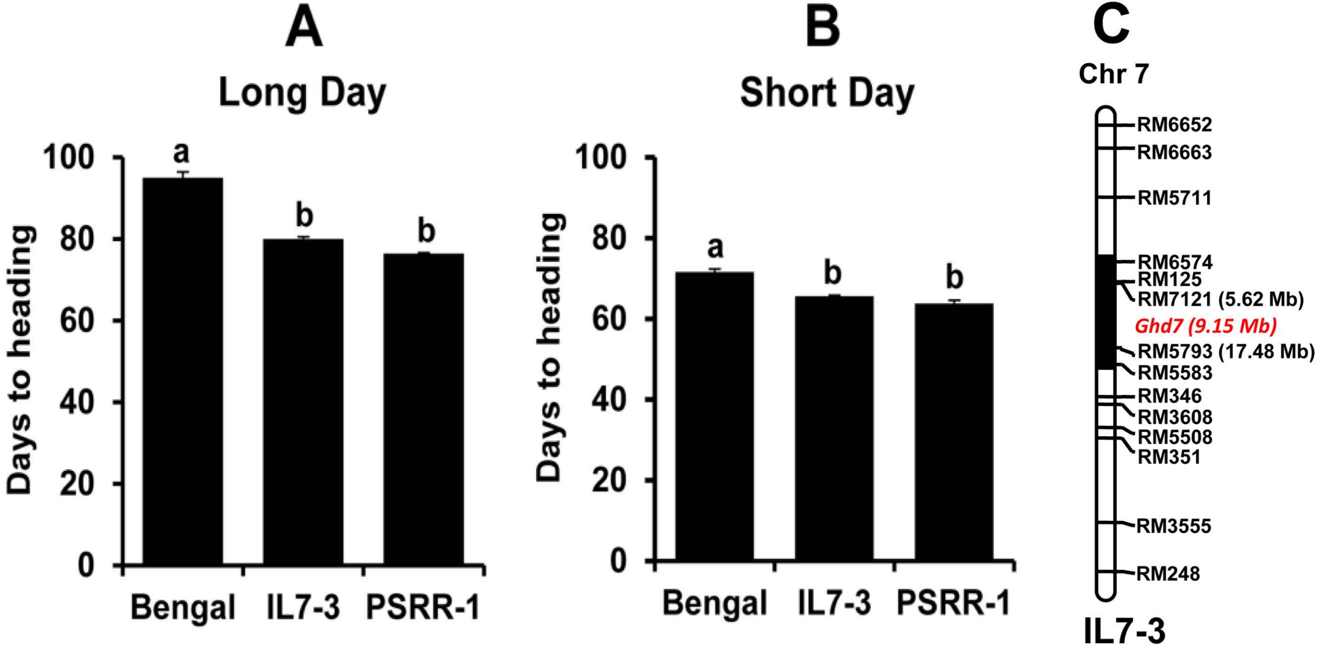

Supplementary Figure S13 Days to heading in IL7-3, 'Bengal', and 'PSRR-1' under long-day (A) and short-day (B) conditions in greenhouse experiments. Planting was done on 16th April, 2015 and July 22, 2015, to expose the plants to long-day and short-day conditions, respectively. One-way analysis of variance was used to determine differences between lines at $P<0.01$. Values are mean ± standard error of five plants per genotype. Graphical genotype of the IL7-3, an IL of PSRR-1 with single introgression indicated by solid black bar on chromosome (C). *Ghd7* (9.15 Mb position on the reference rice genome) is located in the introgressed region of the IL7-3 based on the physical map location. Chromosome map was from the RIL linkage map[34].

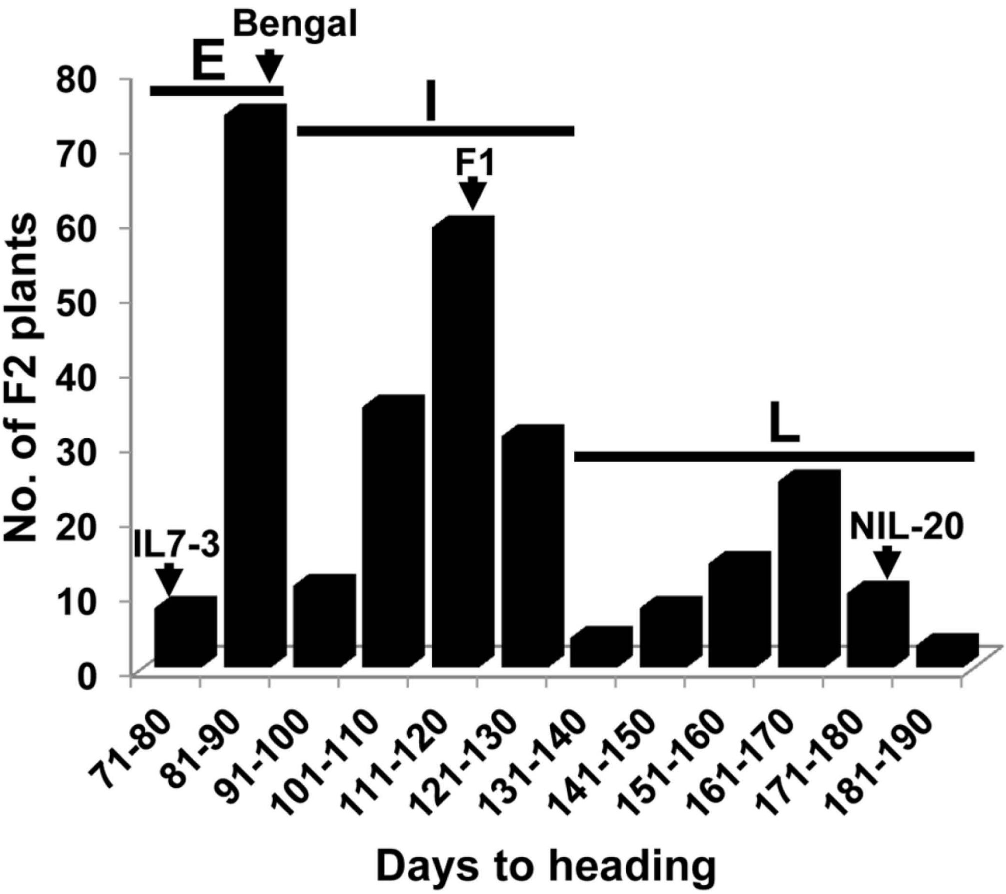

Supplementary Figure S14 Frequency distribution of days to heading in the F_2 population (n=282) derived from the cross BRNIL-20 x IL7-3. Planting was done in mid-April 2014 to expose the plants to natural long-day condition. The F_1 between BRNIL-20 and IL7-3 flowered in 118 days. Days to heading in IL7-3 was significantly different from 'Bengal' due to substitution of the 'Bengal' alleles with 'PSRR-1' allele for the $qHD7\text{-}1^{BR}$. The F_2 plants flowering in ≤90, 91-130, and >130 days were classified as early (E), intermediate (I), and late (L).

BRNIL-20 (AAbb) x BRIL7-3 (aaBB)

(Late Photosensitive) ↓ (Early Photoinsensitive)

F_1 (AaBb) (Intermediate)

↓

F_2

1/16 AABB: Early	1/8 AaBB: Intermediate	1/16 aaBB: Early
1/8 AABb: Late	1/4 AaBb: Intermediate	1/8 aaBb: Early
1/16 AAbb: Late	1/8 Aabb: Intermediate	1/16 aabb: Early

Supplementary Figure S15 Model for genetic interaction between *Hd1* and an unknown locus on chromosome 7 near RM214 for flowering response. *Hd1* alleles of 'PSRR-1' and 'Bengal' were represented as A and a, respectively. The alleles of 'PSRR-1' and 'Bengal' for the unknown gene corresponding to $qHD7\text{-}1^{BR}$ on chromosome 7 were represented as B and b, respectively. Both loci in homozygous condition for 'PSRR' allele were required for photo-insensitivity. The early, intermediate, and late flowering plants in the F_2 population from the cross BRNIL-20 x BRIL7-3 segregated in 5:8:3 ratio (n=282). All late flowering plants were homozygous for 'PSRR-1' *Hd1* allele. Frequency distribution of the F_2 population from this cross was shown in Fig. S14.

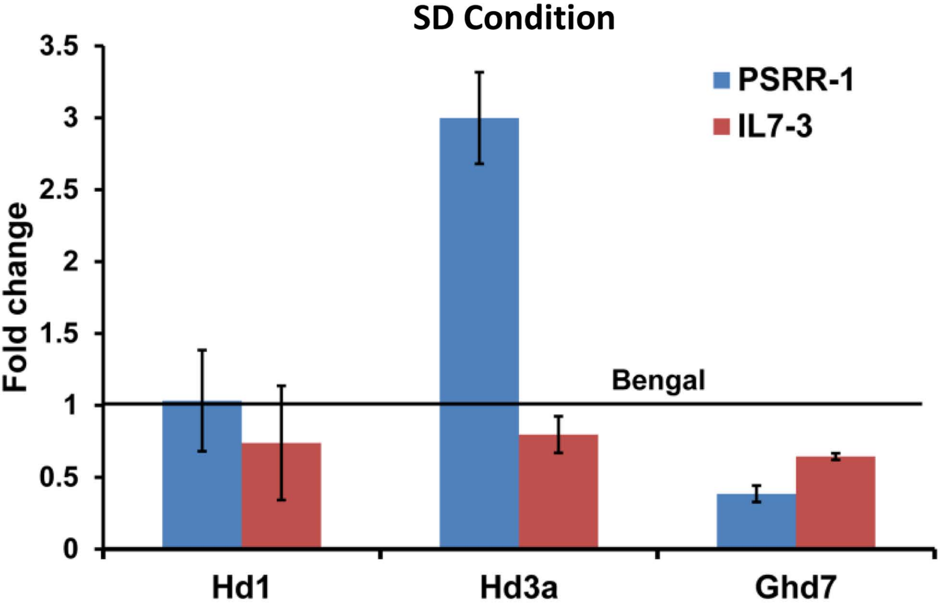

Supplementary Figure S16 Comparative measurements of gene expression of flowering genes (*Hd1, Hd3a,* and *Ghd7)* in leaf tissues of 30 day old seedlings of 'PSRR' and IL7-3, compared to 'Bengal' under short-day conditions. The rice *Actin1* gene was used as the internal control for normalization. The mean values were based on the average of three biological replicates per genotype calculated using relative quantification method[48].

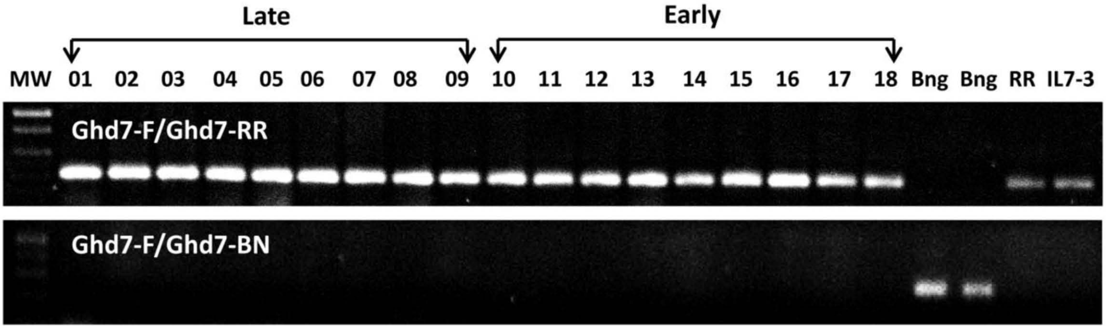

Supplementary Figure S17 Segregation for the *Ghd7* SNP alleles in parents and 18 early and late flowering F_3 progenies derived from the early flowering F_2 plant #229, which was selected from the cross BRNIL-20 x IL7-3. All early and late flowering plants had 'PSRR-1' *Ghd7* allele. Specific targeted alleles were amplified using primers Ghd7-F/Ghd7-RR and Ghd7-F/Ghd7-BN for 'PSRR-1' (RR) and 'Bengal' (Bng) genotypes, respectively.

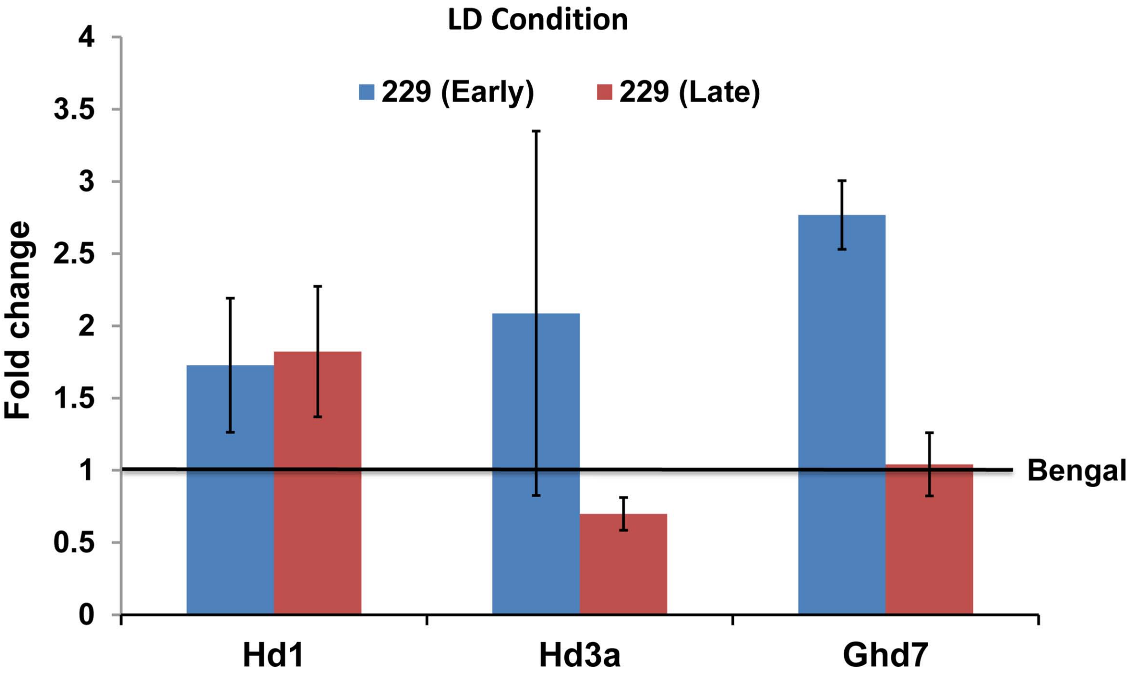

Supplementary Figure S18 Comparative measurements of gene expression of flowering genes (*Hd1, Hd3a,* and *Ghd7*) in leaf tissues of 30 day old early and late flowering F_3 plants from the F_2 plant #229 compared to 'Bengal' under long-day condition. The plant #229 was selected from the F_2 population from the cross BRNIL-20 x IL7-3. The rice *Actin1* gene was used as the internal control for normalization. The mean values were based on the average of three biological replicates per genotype calculated using relative quantification method[48].

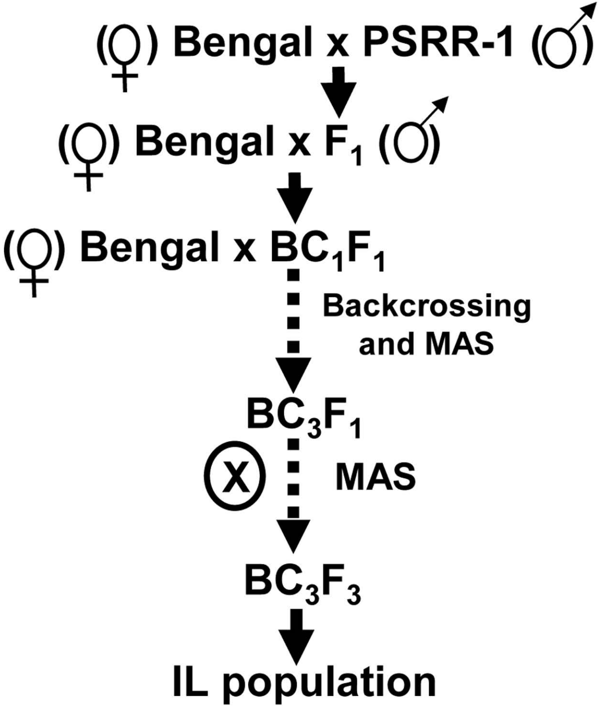

Supplementary Figure S19 A schematic diagram of the development of the introgression lines (ILs) of the weedy rice accession 'PSRR-1' in the cultivated rice 'Bengal' background[34]. MAS: Marker assisted selection.

Supplementary Table S1 Quantitative trait loci, additive effects, and direction of phenotypic effect for days to heading derived from the evaluation of the ILs and the recurrent parent (RP) 'Bengal'. The presence of QTLs was inferred when there was significant difference between the means of each IL and the recurrent parent using Dunnett's test. All ILs significantly different from the recurrent parent at $p < 0.05$ were listed. These data were used to narrow down the QTL regions using substitution mapping. Direction of phenotypic effect (DPE) was denoted by B and R, which indicates either the 'Bengal' and 'PSRR-1' allele increasing the trait values, respectively. The mean days to heading for 'Bengal' and 'PSRR-1' were 76.1 ± 0.4 d and 87.9 ± 1.2 d, respectively. Additive effect (AE) was calculated as follows: Additive effect = (mean of IL-mean of Bengal/2.

IL #	Mean	SE	p-value	Marker interval	Deviation from RP	AE	AE contribution (%)	DPE
2-2	83.0	0.97	0.0016	RM7581-RM145	6.9	3.5	4.5	R
2-3	81.5	2.28	0.0419	RM5512-RM29	5.4	2.7	3.5	R
3-1	83.9	0.59	0.0001	RM3203-RM5819	7.8	3.9	5.1	R
6-1	161.7	0.83	<0.0001	RM469-RM276	85.6	42.8	56.2	R
6-2	146.8	0.65	<0.0001	RM225-RM4924	70.7	35.4	46.5	R
6-3	164.6	0.67	<0.0001	RM111-RM7193	88.5	44.3	58.1	R
7-3	70.30	0.52	0.0194	RM6574-RM5583	-5.8	-2.9	-3.8	B
7-6	69.5	0.43	0.0034	RM3555-RM248	-6.6	-3.3	-4.3	B
7-7	68.8	0.35	0.0011	RM248	-7.3	-3.7	-4.8	B
8-1	83.1	0.46	0.0013	RM408-RM1376	7.0	3.5	4.6	R
10-4	97.8	7.47	<0.0001	RM258-RM333	21.7	10.9	14.3	R
12-4	110.1	0.74	<0.0001	RM7619-RM313	34.0	17.0	22.3	R

Supplementary Table S2 Epistatic QTLs for heading date identified in RIL populations developed from the Bengal x PSRR-1 and Cypress x PSRR-1 crosses[34].

Population	QTL1	QTL2	LOD	Additive effect	$R^{2\,\psi}$
Bengal x PSRR-1	*qHD2-1*^{BR}	*qHD6*^{BR}	1.48	2.026	2.0
	qHD2-2^{BR}	*qHD6*^{BR}	0.74	-1.303	1.0
	qHD6^{BR}	*qHD7-1*^{BR}	23.92	-10.610	14.2
Cypress x PSRR-1	*qHD3*^{CR}	*qHD7*^{CR}	1.057	-3.255	0.9

$^{\psi}$ Phenotypic variation (%) explained by the QTL interaction.

Supplementary Table S3 Identification of putative genetic interactions between a chromosome 6 segment and a chromosome 7 segment for photoperiodic flowering using introgression lines from the BR cross. These three introgression lines had only three substituted 'PSRR-1' segments of which the chromosomes 6 and 7 segments attained homozygosity and chromosome 10 segment (RM1146-RM3451-RM228-RM333) was heterozygous. A and B represent 'Bengal' homozygotes and 'PSRR-1' homozygotes, respectively.

		qHD6^{BR}			***qHD7-1^{BR}***	
**ILs**	**Days to Heading**	**RM 3431**	**RM 4924**		**RM 7121**	**RM5793**
**AE-26-1**	**98**	**R**	**R**		**R**	**R**
**AE-26-2**	**101**	**R**	**R**		**R**	**R**
**AE-26-3**	**>200**	**R**	**R**		**B**	**B**
**Bengal**	**85**	**B**	**B**		**B**	**B**

Supplementary Table S4 Validation of genetic interactions between *qHD6*^{BR} and *qHD7-1*^{BR} involved in photoperiodic flowering in the BR-RIL population[34]. The genotypic profiles of highly photosensitive late flowering RILs and very early flowering RILs were compared for both chromosomal regions harboring the QTLs. The *qHD6*^{BR} corresponded to the *Hd1* locus. The nearest markers for *qHD6*^{BR} and *qHD7-1*^{BR} were RM3431 and RM214, respectively. A and B represent 'Bengal' homozygotes and 'PSRR-1' homozygotes, respectively. *Rc* is a morphological marker for red pericarp.

**Late flowering photosensitive RILs**									
			***qHD6*^{BR}**				***qHD7-1*^{BR}**		
**RIL #**	**Days to Heading**		**RM276**	**RM3431**	**RM4924**		***Rc***	**RM214**	**RM5793**
26	156.0		R	R	R		B	B	B
77	168.0		R	R	R		B	B	B
3	136.0		R	R	R		B	B	R
19	137.0		B	R	R		B	B	B
32	138.0		R	R	R		B	B	B
39	155.0		R	R	R		B	B	R
42	147.0		R	R	R		B	B	B
53	163.0		R	R	R		B	B	B
60	139.0		R	R	R		B	B	B
120	159.0		R	R	B		B	B	R
**Early flowering RILs**									
			***qHD6*^{BR}**				***qHD7-1*^{BR}**		
**RIL #**	**Days to Heading**		**RM276**	**RM3431**	**RM4924**		***Rc***	**RM214**	**RM5793**
15	85.0		R	R	R		R	R	R
17	89.0		R	R	R		R	R	R
27	80.0		R	R	R		R	R	R
29	92.0		R	R	R		R	R	R
30	92.0		R	R	R		R	R	R
31	79.0		R	R	R		R	R	R
33	71.0		R	R	R		R	R	R
44	71.0		R	R	R		B	R	R
50	77.0		R	R	R		R	R	R
51	71.0		R	R	R		R	R	R
57	71.0		R	R	R		R	R	R
63	93.0		B	R	R		R	R	R
72	76.0		R	R	R		R	R	R
75	93.0		R	R	R		R	R	B
79	91.0		R	R	R		R	R	R

84	92.0		R	R	R		R	R	B
86	71.0		R	R	R		R	R	R
90	95.0		R	R	R		R	R	R
93	69.0		R	R	R		R	R	R
100	72.0		R	R	R		R	R	R
111	79.0		-	R	R		R	R	R
115	85.0		B	R	R		R	R	R
116	100.0		R	R	R		R	R	R
122	89.0		-	R	R		R	R	R
131	85.0		R	R	R		R	R	B
134	83.0		R	R	R		R	R	R
139	95.0		R	R	R		R	R	R
155	77.0		R	R	R		R	R	R
156	78.0		R	R	R		R	R	R
158	89.0		R	R	R		R	R	R
169	75.0		B	R	R		R	R	R
172	89.0		R	R	R		R	R	R
175	93.0		R	R	R		R	R	R
177	91.0		R	R	R		R	R	R
178	93.0		R	R	R		R	R	R
180	71.0		R	R	R		R	R	R
183	89.0		R	R	R		R	R	R
184	77.0		R	R	R		R	R	R
189	100.0		R	R	R		R	R	R
191	77.0		B	R	R		R	R	R
194	71.0		R	R	R		R	R	R
195	89.0		B	R	R		R	R	B
9	85.0		R	R	R		B	R	R
11	73.0		R	R	R		R	R	R
43	71.0		R	R	R		B	R	R
65	94.0		R	R	R		R	R	R
68	73.0		R	R	R		B	R	R
73	91.0		R	R	R		R	R	B
103	89.0		R	R	R		R	R	R
130	89.0		R	R	R		R	R	B
140	89.0		R	R	R		R	R	R
151	89.0		R	R	R		R	R	R

Supplementary Table S5 Average day length (A) and temperature (B) in Baton Rouge, Louisiana, USA in 2009, 2011, 2013, 1014, and 2015 (Source: http://www.usclimatedata.com/climate/baton-rouge/louisiana/united-states/usla0033).

A. Average day length (hours) between January and December.

Month/Year	2009	2011	2013	2014	2015	Average
Jan	10:26:38	10:26:08	10:26:41	10:26:26	10:26:11	10:26:25
Feb	11:07:50	11:07:02	11:07:54	11:07:29	11:07:05	11:07:28
Mar	12:00:39	11:59:46	12:00:42	12:00:15	11:59:48	12:00:14
Apr	12:55:55	12:55:05	12:55:57	12:55:32	12:55:07	12:55:31
May	13:42:09	13:41:33	13:42:10	13:41:52	13:41:34	13:41:52
Jun	14:04:58	14:04:51	14:04:57	14:04:54	14:04:50	14:04:54
Jul	13:53:02	13:53:28	13:52:59	13:53:12	13:53:26	13:53:14
Aug	13:12:24	13:13:10	13:12:21	13:12:44	13:13:07	13:12:45
Sep	12:19:35	12:20:27	12:19:32	12:19:58	12:20:24	12:19:59
Oct	11:24:39	11:25:30	11:24:37	11:25:02	11:25:28	11:25:03
Nov	10:37:05	10:37:43	10:37:04	10:37:23	10:37:42	10:37:23
Dec	10:13:02	10:13:09	10:13:02	10:13:06	10:13:09	10:13:06

B. Average temperature (Celsius) between January and December.

Month/Year	2009	2011	2013	2014	2015	Average
Jan	12.52	9.17	12.47	6.94	9.42	10.10
Feb	14.58	12.22	13.22	11.61	9.89	12.30
Mar	18.14	17.67	13.56	14.72	18.39	16.49
Apr	20.05	21.86	18.94	20.33	21.89	20.61
May	24.58	23.94	22.56	23.33	24.67	23.81
Jun	28.42	28.83	27.5	26.94	27.39	27.81
Jul	28.81	28.75	27.39	27.28	29.61	28.36
Aug	27.75	30.19	27.67	28	28.44	28.41
Sept	26.22	24.39	27.06	26.44	26.36	26.09
Oct	20.69	18.69	21.22	21.28	21.81	20.73
Nov	14.81	15.81	13.89	12.56	18.06	15.02
Dec	10.28	12.33	11.17	13.33	15.56	12.53

Supplementary Table S6 List of primers used in this study.

(A) Primers for qRT-PCR experiments

**Gene name**	**Forward primer (5'-3')**	**Reverse primer (5'-3')**
Hd1	AGGGCCGTTTCGCCAAGAGA	ACCGTCAGATAGAGCTGCAGTGGA
Hd3a	AGCCCAAGTGACCCTAACCT	GTTGTAGAGCTCGGCGAAGT
RFT1	AGGATTGTGGGTGATGTGCT	ATTGGAGACGATCCTTGCAC
Ehd1	GCGCTTTTGATTTCCTGC	TTCGGAATATGTGCTGCC
Ehd2	TCGATCCTCCATGTTGTTGA	GCTTCTTGGCATCCAGAGTC
OsGI	CCTGCTGAACTTGCAAATGA	TACAGATGATCCACCGCTGA
Ghd7	GATGATGGGGAGAGCTTGAA	CATCTCGGCATAGGCTTTTC
Hd6	TCCAAGTGAGGGCTGCGGAGA	ACGGTGCAGCTCCATGACTGC
ETR2	CTCCTGGAGAAGCTTGGTTG	AACCACCAGCTGGAAAGATG
Oslhy	AGCAGGATCGCTCTTCATGT	AGATGCTTGCTTTGCCTTGT
OsActin	ATCCTTGTATGCTAGCGGTCGA	ATCCAACCGGAGGATAGCATG

(B) primers for *Hd1* gene and *Hd3a* promoter sequencing

**Fragment name**	**Forward primer (5'-3')**	**Reverse primer (5'-3')**
Hd1_Genomic_F1	TGCGAGGTAGAGGAACAGGAGAAG	TCCCTTCCTTCTCTGCAAACTCC
Hd1_Genomic_F2	CATGTATTTTGGTGAAGTCGATGAG	GAGACCCTGCAATGATATATAGATGG
Hd1_Genomic_F3	GTACACAGCAATCACCACACGAAAG	CCCCAATTCGTCACCTAAACCC
Hd1_CDS_F1	TGCGAGGTAGAGGAACAGGAGAAG	CTCAGCGAGGACGGAGGTG
Hd1_CDS_F2	CAGGTGCACTCCGCGAAC	CTGATGGAATCTGTGTAAGCACTGAC
Hd1_CDS_F3	GTACCTTCACAGATCACAATGCTGAG	TCAGAACCATGGAACAGTACCATAGCT
Hd3a_promoter1	GTTCCGGATATATGCCCCTCCGG	CCATTGGACACGGTCTTGGAG
Hd3a_promoter2	CATTAATTGCCTTACCTCAAC	CGATCTTGCAAAAAACCCTG
Hd3a_Seq	CGGTTCCCTTTAAAAATTAGAAG	GAGCTAGCTAGCTATCTAGC

(C) Hd1 deletion primers for *Hd1* genotyping

**Primer name**	**Forward primer (5'-3')**	**Reverse primer (5'-3')**
Hd1-del_	CACCTCCGTCCTCGCTGAGG	CTCTTGCTGTTCATGCATCCCATACTGCTG

(D) Ghd7 SNP primers

Ghd7-F: 5'-TATTTGACCTTTGATAGTTTGAAGTTGTACGGCATA-3'

Ghd7-BN-R, 5'-ACGTTCGACGTCTCCCTCGGCTA-3'

Ghd7-RR-R, 5'-CGTTCGACGTCTCCCTCGTCGG-3'
